# Simulation and performance analysis of a solar-assisted venturi-plasma pasteurization system for milk

**DOI:** 10.1371/journal.pone.0352384

**Published:** 2026-07-02

**Authors:** Kimia Taki, Bahram Hosseinzadeh Samani

**Affiliations:** Department of Mechanical Engineering of Biosystem, Shahrekord University, Shahrekord, Iran; University of Shanghai for Science and Technology, CHINA

## Abstract

This research evaluates the feasibility and performance of a novel solar-powered milk pasteurization system integrating a Venturi tube hydrodynamic reactor and liquid-phase cold plasma technology. Designed for a daily capacity of 600 liters, the system utilizes flat-plate collectors and photovoltaic panels to supply thermal and electrical energy, respectively. Performance was simulated across three Iranian cities with distinct climates: Shahrekord (cold, mountainous), Yazd (hot, arid), and Bandar-e-Abbas (hot, humid). The results revealed that annual efficiencies for solar collectors and PV panels reached up to 0.47 and 0.18, respectively. Yazd exhibited the highest solar energy potential with a peak radiation of 8311 kWh/m^2^, while Shahrekord demonstrated the most consistent thermal gain during spring and summer. The system successfully maintained the target pasteurization temperature (40 ± 5°C) with a solar fraction ranging from 0.036 to 0.32. These findings confirm that the integrated system reduces dependence on fossil fuel-based energy by increasing renewable energy contribution up to 32% (solar fraction) and also provides a promising sustainable solution for remote and energy constrained regions for milk pasteurization in diverse climatic conditions, enhancing both operational efficiency and environmental sustainability.

## 1. Introduction

Milk is a critical food source providing essential nutrients, but its susceptibility to microbial contamination necessitates pasteurization to ensure safety and extend shelf life [1,2]. Traditional pasteurization processes are energy-intensive, leading to high economic costs and environmental impacts from fossil fuel dependency. In remote or energy-constrained regions, limited access to electricity exacerbates these challenges, increasing milk waste and operational cost [3–5]. These constraints highlight the necessity of innovative non-thermal pasteurization methods that can operate efficiently alongside renewable energy systems.[1, 2] Traditional pasteurization processes are energy-intensive, leading to high economic costs and environmental impacts from fossil fuel dependency. In remote or energy-constrained regions, limited access to electricity exacerbates these challenges, increasing milk waste and operational cost [3–5]. These constraints highlight the necessity of innovative non-thermal pasteurization methods that can operate efficiently alongside renewable energy systems.

Solar energy offers a clean, abundant, and accessible renewable alternative for pasteurization, particularly in sunny regions like Iran. However, precise temperature control and thermal stability requirements pose technical challenges for solar applications in this process.

Solar radiation intensity, ambient temperature, humidity, and climatic variability significantly affect the thermal and electrical performance of solar-assisted systems. Previous studies have demonstrated that photovoltaic efficiency is strongly dependent on solar irradiance and module operating temperature, while environmental conditions such as humidity and atmospheric scattering can considerably influence the energy output and long-term stability of PV systems. In addition, variations in solar radiation and climatic conditions directly impact the thermal efficiency of solar collectors and the overall reliability of hybrid solar energy systems. Therefore, evaluating solar-assisted pasteurization systems under different climatic conditions is essential for achieving stable thermal performance and efficient renewable energy utilization [6–8].

Previous studies have primarily focused on solar heating systems, with limited research on milk pasteurization. However, most existing works rely on conventional thermal approaches, and only a few studies specifically address solar-assisted milk pasteurization, indicating a clear research gap that the present study aims to fill. For instance, Sur et al. (2020) developed a 150-liter solar system using parabolic collectors, achieving low-temperature long-time (LTLT) pasteurization at 75°C for 30 minutes in remote areas [9]. In another study, conducted by Yaseen et al. (2019), reported improved thermal efficiency and reduced costs (1.8 Rs/liter) with a similar LTLT solar pasteurizer [10]. Other works highlight solar systems’ potential for small-scale producers in rural settings, minimizing energy consumption and waste [11]. Hybrid approaches, such as PV/T systems combined with heat pumps, have shown up to 8.8% energy savings per kg of milk [12].

These studies demonstrate solar pasteurization’s feasibility but often rely on conventional thermal methods. This research introduces a novel non-thermal approach combining hydrodynamic cavitation using Venturi tube with liquid-phase cold plasma for enhanced microbial inactivation. Unlike previous solar pasteurization studies that rely solely on thermal mechanisms, this synergistic method enables improved microbial inactivation by allowing cavitation to enhance radical dispersion, turbulence, and mass transfer generated by liquid-phase plasma.

The Venturi tube induces hydrodynamic cavitation, where pressure drops create bubble collapse, generating agitation, mass transfer, and cell disruption [13,14].[13,14].

Liquid-phase cold plasma, formed by high-voltage discharge in the liquid medium, produces reactive species (radicals, ions) with superior penetration for disinfection compared to gas-phase plasma [15,16].

By integrating these technologies, cavitation enhances radical distribution and turbulence, improving inactivation efficiency. This study designs and simulates a solar-assisted system providing thermal preheating (flat-plate collectors) and electrical power (PV panels) for a 600 l/day pasteurization unit (equivalent to a 30-cow farm). While non-thermal technologies such as hydrodynamic Venturi reactors and cold plasma significantly reduce the thermal degradation of milk nutrients, their integration with solar energy systems offers a dual advantage. Firstly, solar thermal collectors provide the necessary pre-heating to optimize the physical properties of the milk (reducing viscosity), which enhances the cavitation efficiency within the Venturi tube. Secondly, utilizing photovoltaic (PV) units ensures that the electrical demand of the cold plasma reactor is met through sustainable and renewable sources, making the system viable for decentralized and remote dairy processing where grid electricity is unreliable or absent.

**Table 1:** Angle ranges for solar collectors and panels across different cities.

Location	Angle range
Shahrekord	(17˚-47˚)
Yazd	(16.90˚-46.90˚)
Bandar-e-Abbas	(12.20˚-42.20˚)

**Table 2:** Monthly and annual optimization of tilt angles for solar panels and collectors in Shahrekord using the RSM method.

Time	Collector Tilt	Collector Efficiency	Panel Tilt	Panel Efficiency
January	47	0.3461	17	0.1878
February	44.91	0.3049	17	0.1870
March	39.02	0.3848	17	0.1854
April	25.78	0.3930	47	0.1847
May	17.83	0.4111	47	0.1842
June	18.63	0.4956	47	0.1832
July	17.77	0.4896	47	0.1828
August	17.48	0.4998	47	0.1826
September	31.24	0.4866	47	0.1826
October	36.84	0.4773	17	0.18385
November	47	0.3912	17	0.1861
December	47	0.3712	17	0.1869
Yearly	22.62	0.4027	47	0.1842

**Table 3:** Monthly and annual optimization of tilt angles for solar panels and collectors in Yazd using the RSM method.

Time	Collector Tilt	Collector Efficiency	Panel Tilt	Panel Efficiency
January	46.90	0.3986	16.90	0.1864
February	46.90	0.5294	16.90	0.1843
March	36.16	0.4159	16.90	0.1840
April	21.76	0.4348	46.90	0.1835
May	16.90	0.4683	46.90	0.1828
June	16.90	0.4889	46.90	0.1823
July	16.90	0.5201	46.90	0.1818
August	17.14	0.5685	46.90	0.1817
September	29.79	0.5579	46.90	0.1816
October	44.54	0.4812	16.90	0.1831
November	46.45	0.4232	16.90	0.1850
December	46.90	0.4197	16.90	0.1860
Yearly	17.24	0.4262	46.90	0.1832

While several non-thermal methods have been studied, they often face practical limits in dairy processing. For example, UV-C has low penetration in opaque milk, and PEF systems often suffer from electrode fouling. To address these issues, a hybrid Venturi-plasma system was selected in this study. The Venturi reactor creates intense turbulence and pressure changes that weaken microbial structures. By generating plasma directly inside the liquid, the system ensures that reactive species are distributed throughout the milk by the turbulent flow, leading to a more uniform disinfection. Therefore, the objective of this research is to evaluate the efficiency of this solar-assisted Venturi-plasma system.

Performance is evaluated in three Iranian cities with diverse climates: Shahrekord (cold, mountainous), Yazd (hot, arid), and Bandar Abbas (hot, humid). The objectives are to optimize system components, assess energy performance (solar fraction, efficiencies), and demonstrate feasibility for sustainable, cost-effective milk pasteurization in energy-limited regions, reducing fossil fuel reliance and greenhouse gas emissions. Overall, this combination of a non-thermal plasma–cavitation pasteurization method with a dual-source solar energy system represents a novel contribution that addresses the key limitations identified in previous studies.

## 2. Materials and methods

### 2.1. Solar pasteurization system

This study is dedicated to the design and feasibility of an integrated system for milk pasteurization in a small production unit with a daily milk input capacity of 600 liters (equivalent to the production of a dairy farm with 30 cows and an average milk production of 20 liters per cow).

This system comprises a solar array to provide the thermal and electrical energy required for the pasteurization process, as well as a pasteurization unit based on a Venturi tube reactor integrated with liquid-phase cold plasma technology. The system’s performance was simulated using a professional transient simulation program. The feasibility of implementing this system in three cities with varying climates was evaluated, including Shahrekord (a cold and mountainous climate), Yazd (a hot and dry climate), and Bandar-e-Abbas (a hot and humid coastal climate).

**Fig 1:**
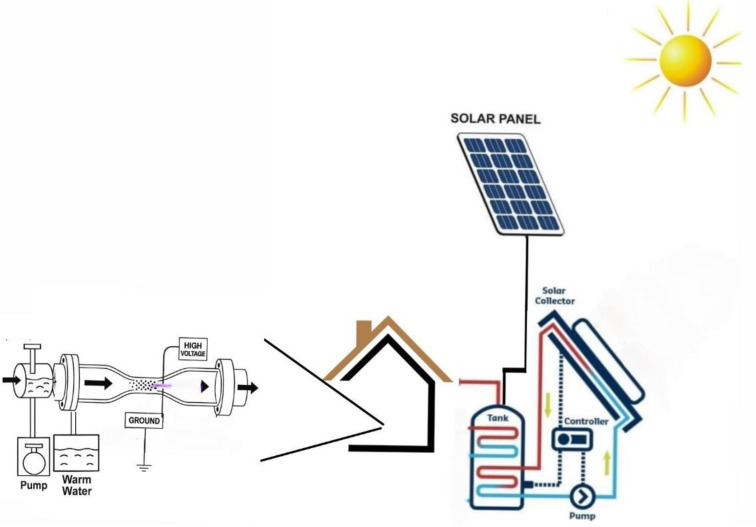
Solar pasteurization system.

According to Fig 1, this system consists of a workshop in which the combined pasteurization system of Venturi tube and cold plasma is installed, and also the elements of electrical and thermal energy supply of this system include a flat plate collector, a photovoltaic panel, a hot water tank, a pump, and a controller. The following sections explain each part of this system separately.

### 2.2. Combined Venturi tube-liquid phase cold plasma pasteurization system

The milk pasteurization system designed in this study utilizes a combination of hydrodynamic Venturi tube technology and liquid phase cold plasma. This system, employing a novel method, enables the effective removal of microorganisms from milk. The special geometry of the Venturi tube reactor creates the necessary conditions for the occurrence of hydrodynamic cavitation. The implosion of cavitation-induced bubbles releases significant energy, which leads to increased molecular mobility and damage to the cell walls of microorganisms. At the same time, the cold plasma produced in the liquid phase is applied to all layers of fluid (milk) passing through the reactor.

This process, combined with cavitation, helps the free radicals generated by the plasma spread throughout the length of the reactor and significantly increases the contact surface between the radicals and microorganisms by creating turbulence, agitation, and effective mass transfer. This combination improves the efficiency of the microbial inactivation process.

### 2.3. Pasteurization and climate feasibility workshop

The pasteurization system is designed and installed in a production workshop with an area of 100 m^2^ and a height of 3 m. This workshop is designed to produce 600 liters of pasteurized milk per day. A feasibility study was conducted for the establishment and operation of this workshop in three Iranian cities with varying climates: Shahrekord (mountainous), Yazd (hot and dry), and Bandar-e-Abbas (humid and coastal). This assessment included examining climatic conditions and their impact on the performance of the solar system and the pasteurization process.

### 2.4. Hydrodynamic Venturi tube reactor

The geometry of the hydrodynamic Venturi tube reactor consists of a simple design with a converging section (throat) and a diverging section (diffuser). According to Bernoulli’s principle, the velocity of the fluid should increase as it passes through this bottleneck, while its static pressure decreases.

**Fig 2:**
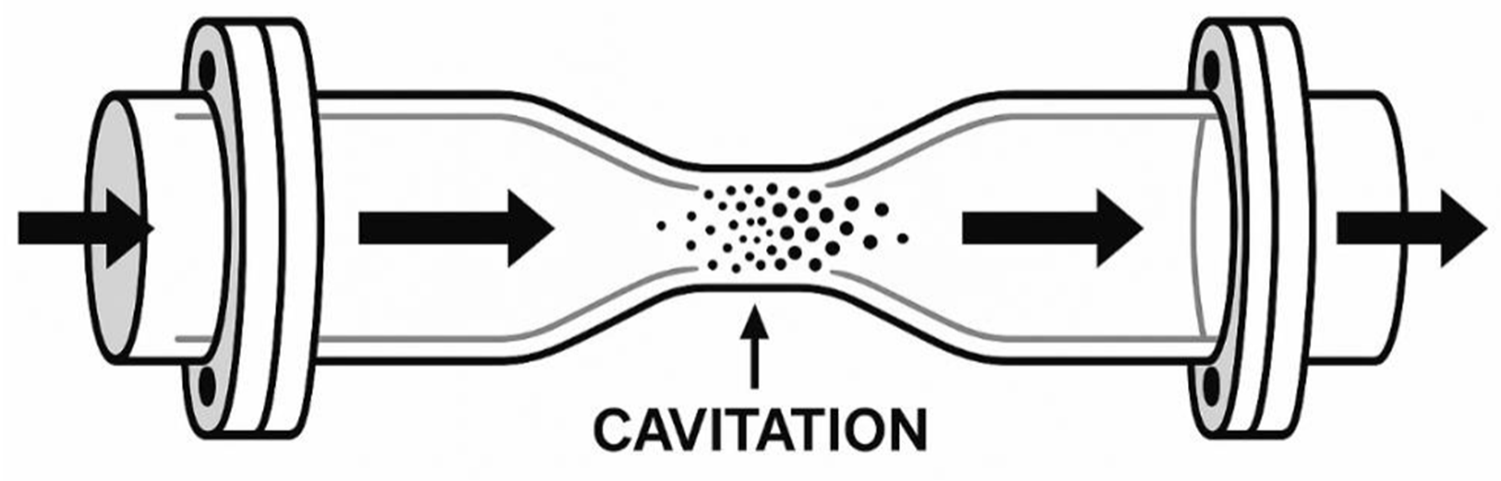
Schematic of hydrodynamic Venturi tube cavitation.

The hydrodynamic cavitation phenomenon is created by the pressure drop resulting from the change in the velocity of the liquid flow inside the reactor. In fact, the phenomenon of cavitation is a physical phenomenon in which the pressure of a liquid decreases and approaches the pressure of saturated water vapor, causing local evaporation of the liquid. During this reaction, bubbles form in the reactor throat. When these bubbles reach high-pressure points (divergent part of the reactor), an explosion occurs. This explosion leads to the release of energy, mixing and stirring, and also the destruction of the colony of microorganisms inside the liquid. Fig 2 shows a hydrodynamic Venturi tube cavitation reactor.

### 2.5. Liquid phase cold plasma system

Liquid-phase plasma refers to the direct delivery of energy into a liquid medium, generating plasma within the liquid. Liquid-phase plasma provides the energy required to break molecular bonds directly through a high-voltage and frequency electrical discharge applied to the reacting liquid, creating a plasma channel between two electrodes in the liquid medium.

**Fig 3:**
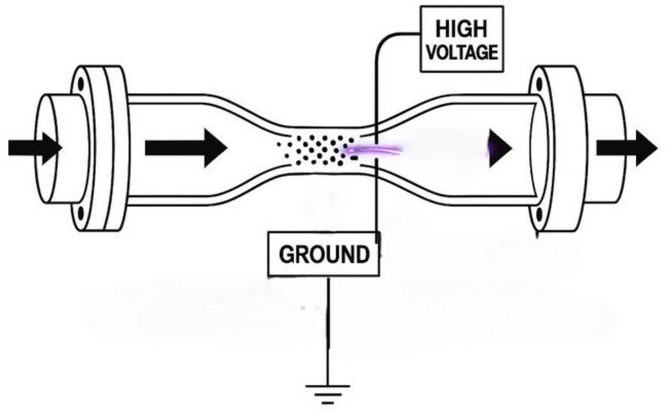
Combined system of a hydrodynamic Venturi tube -liquid phase plasma reactor.

This system consists of two coaxial electrodes installed at a specified distance from each other in the divergent part of the venturi tube (diffuser). Considering that the melting temperature of tungsten exceeds 3000˚C and it does not melt when subjected to high voltage, the material of the electrodes is selected to be tungsten. One of the electrodes is connected to the high-voltage source, and the other electrode is the ground electrode (Fig 3).

### 2.6. Simulation of the solar pasteurization system

**Fig 4:**
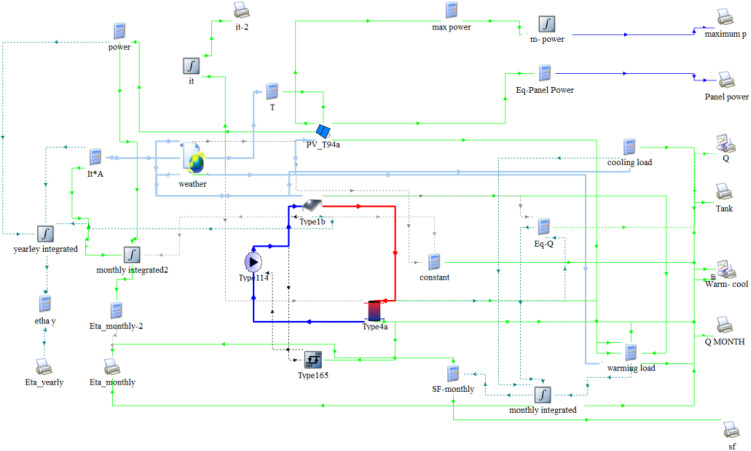
Computer simulation of the solar pasteurization system.

To evaluate the long-term performance of the solar pasteurization system, a dynamic simulation was carried out using a professional transient simulation program widely used in solar thermal and photovoltaic system studies. The simulation was performed with one-hour time steps over one year. This system consists of two subsystems to provide the energy required for the pasteurization process: (1) a thermal subsystem, consisting of a solar water heater and related components, which provides the required thermal energy; and (2) an electrical subsystem, consisting of photovoltaic panels, which provides the electrical energy for the system. The following section introduces and explains the components of this system, along with the equations governing them (Fig 4).

### 2.7. Main assumptions and key simulation parameters

The dynamic performance of the integrated solar pasteurization system was simulated in computer software over one typical meteorological year using an hourly time step (8760 time steps).

The main modeling assumptions and key parameters are summarized below:

- The milk flow rate through the Venturi–plasma reactor was assumed constant and equivalent to the design processing capacity of 600 L day^−1^.
- The 500 L stratified storage tank was controlled at a hot‑zone temperature of 40 ± 5 °C using an on/off control strategy.
- Overall heat loss coefficients (UA values), derived from manufacturer data and literature, were applied to the flat‑plate collector, storage tank, and piping network.
- A fixed‑speed circulation pump with a nominal mass flow rate of 1000 kg h^−1^ was modeled, operating in on/off mode based on the collector outlet–inlet temperature difference.
- The workshop space (100 m^2^ floor area, 3 m height) was maintained at 24 °C with 10 air changes per hour.
- The PV modules were modeled using the single‑diode model, while the flat‑plate collector performance was calculated using standard efficiency equations.
- Auxiliary electric heating was activated only when the storage tank temperature fell below the defined deadband.
- Secondary effects, including collector fouling, long‑term PV degradation, and temperature‑dependent variations in milk thermophysical properties, were neglected to simplify the transient simulation.

These assumptions are consistent with standard practices in transient solar thermal and photovoltaic simulations and enable transparent and reproducible assessment of system performance under varying climatic conditions.

Although a conventional on/off control strategy was employed in the present simulation to regulate the storage tank temperature within the defined deadband (40 ± 5 °C), previous studies have reported the application of advanced predictive control approaches, such as nonlinear model predictive control (NMPC), in thermal management systems to improve energy efficiency and operational stability under variable environmental conditions. Therefore, the integration of predictive control strategies may provide an effective approach for future optimization of auxiliary energy management and solar energy utilization in hybrid solar-assisted systems [17].

### 2.8. Analysis, modeling, and optimization of the experiment

To optimize the collector and solar panel angles in the solar pasteurization system, Design-Expert software and the Response Surface Methodology (RSM) were employed. This approach, through mathematical and statistical modeling, determines the relationship between the independent variables (collector and panel angles) and the dependent variables (monthly and annual efficiency). In this study, optimization was performed for three different cities. For each city, four separate optimizations were conducted as follows:

1. The collector angle is the independent variable, and monthly efficiency is the dependent variable (one for each month).
2. The panel angle is the independent variable, and monthly efficiency is the dependent variable (one for each month).
3. The collector angle is the independent variable, and annual efficiency is the dependent variable.
4. The panel angle is the independent variable, and annual efficiency is the dependent variable.

For each optimization, a one-factor design was used. By specifying the minimum and maximum angles of the collector and panel (based on the geographical conditions of each city), the number and levels of the experiments were determined. The mathematical model used for optimization is represented by Equation (1).


$$\begin{array}{c} Yi=\beta_{\text{0}}\;+\sum \beta_{i}X_{i}+Y_{i}\text{=}\beta_{\text{0}}+\sum \beta_{i}X_{i}\;+\sum \beta_{ij}X_{i}X_{j}+\sum \beta_{jj}X_{i2}+\varepsilon \end{array}$$ (1)

Where β_0_, β_i_, β_ij_, and β_jj_ are fixed coefficients, X_i_ and X_j_ are independent process variables, ɛ: random error [18].

Table 1 shows the range of each independent variable separately for each city

**Table 5:** Average and standard deviation of photovoltaic panel performance.

Cities	Maximum Photovoltaic panel power (kWh)	Month	Minimum Photovoltaic panel power (kWh)	Month
Shahrekord	712	September	19.10	February
Yazd	712	March	25.10	February
Bandar-e-Abbas	672	April	20.2	February

## 3. Results and discussion

The milk pasteurization process holds particular importance due to its vital role in ensuring food safety and its direct connection to human health. Given the energy-intensive nature of this process and the need to reduce dependence on fossil fuels, a hybrid solar pasteurization system was designed and simulated in this study. This system utilizes flat plate collectors to supply the required thermal energy and photovoltaic panels to generate the electrical energy needed for a pasteurization facility with a daily capacity of 600 liters of milk.

The system’s performance was simulated in three Iranian cities with distinct climates: Shahrekord (a cold, mountainous climate), Yazd (a hot, arid climate), and Bandar-e-Abbas (a hot, humid coastal climate). The simulations spanned one year (from January 1, 2024, to December 31, 2024), with one-hour time steps, to comprehensively assess the impact of climatic conditions on system performance.

The following results analyze the performance of the solar pasteurization system across three cities, Shahrekord, Yazd, and Bandar-e-Abbas, based on the methodologies described (computer software simulations and RSM optimization). Key metrics (including useful energy (Qu), energy to load (Ql), auxiliary energy (Qaux), and solar fraction (SF)) are evaluated to assess system efficiency and climatic impacts, building on the computational framework outlined in Section 2. Also, in this section, the simulation results are analyzed and compared to examine the relationship between solar radiation and the system outputs. Furthermore, the collector and photovoltaic panel angles were optimized using the Response Surface Methodology (RSM) with Design-Expert software to determine the optimal angles for maximizing monthly and annual efficiency in each city.

### 3.1. Optimization of collector and solar panel angles in the pasteurization system of Shahrekord

Shahrekord, the capital of Chaharmahal and Bakhtiari Province, is situated at an elevation of approximately 2,060 meters above sea level and has a cold, mountainous climate characterized by cold winters and mild summers. These conditions provide a suitable context for utilizing solar energy.

The city’s average annual solar irradiation is around 18,500 kJ/m^2^/day (equivalent to 5.14 kWh/m^2^/day), offering significant potential for a solar pasteurization system. Adjusting the tilt angles of flat-plate collectors and photovoltaic panels to maximize solar irradiation in different seasons enhances the system’s thermal and electrical efficiencies, reducing dependence on auxiliary energy sources, such as electric heaters.

In this study, to determine the optimal angles for achieving the highest monthly and annual efficiencies, Design-Expert software, the response surface methodology (RSM), and a one-factor model were employed. The optimization results for the collector and panel angles in Shahrekord are presented in Table 2. For the flat-plate collector, the optimal tilt angle ranged from 39.02° to 47° during the cold months and from 17.48° to 25.78° in the warm months. The collector efficiency peaked in summer (particularly August) at 0.4999, consistent with the high solar irradiation during this period. Conversely, efficiency dropped to 0.30494 in winter (February), indicating the impact of reduced irradiation and the potential need for auxiliary heating during the colder months.

For the photovoltaic panels, the optimal angle was determined to be 17° during the cold months (January to March and October to December) and 47° during the warm months (April to September). These angles align well with the sun’s position in the sky throughout the year. A lower angle in winter optimizes the panels for capturing the low-altitude sun, while a higher angle in summer positions them nearly perpendicular to the incoming radiation. The panel efficiency varied between 0.1826 and 0.1878, demonstrating stable performance to supply power to the pump and cold plasma reactor.

A study conducted in China across six cities with different climates highlighted the influence of latitude and climate on the optimal angle. One of these cities, Lhasa, which has a cold, high-altitude climate similar to Shahrekord, reported an optimal annual collector tilt of 27.5°, closely matching the optimal annual angle obtained for Shahrekord [19]. Additionally, Pantic et al. (2016) reported in Serbia that temperatures below 20 °C in December increased photovoltaic efficiency to 16%, whereas temperatures of 55 °C in August reduced it to 10% [20]. This supports the finding that Shahrekord’s cold winter temperatures facilitate high panel efficiency (0.1878 in January), while the optimal tilt of 47° in summer ensures stable efficiencies ranging from 0.1826 to 0.1847. These results highlight the importance of adjusting panel angles to maintain electrical performance and achieve the target pasteurization temperature of 40 ± 5 °C.[19]. Additionally, Pantic et al. (2016) reported in Serbia that temperatures below 20 °C in December increased photovoltaic efficiency to 16%, whereas temperatures of 55 °C in August reduced it to 10% [20]. This supports the finding that Shahrekord’s cold winter temperatures facilitate high panel efficiency (0.1878 in January), while the optimal tilt of 47° in summer ensures stable efficiencies ranging from 0.1826 to 0.1847. These results highlight the importance of adjusting panel angles to maintain electrical performance and achieve the target pasteurization temperature of 40 ± 5 °C.

The annual efficiencies of the collector and panels were found to be 0.4028 and 0.1842, respectively, aligning well with the values predicted by the optimization model. These findings highlight the crucial role of angle adjustment in maintaining a pasteurization temperature of 40 ± 5°C and minimizing operational costs in the cold climate of Shahrekord.

**Fig 5:**
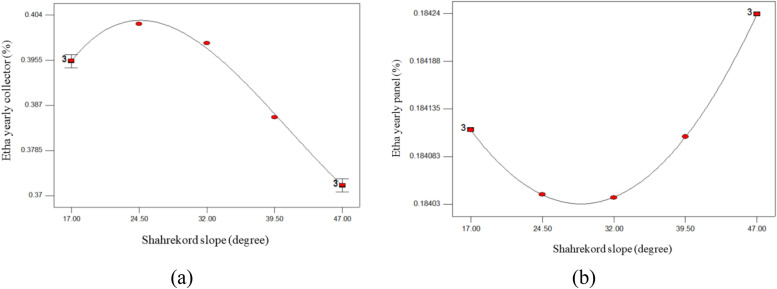
(a) Optimal collector slope, (b) optimal photovoltaic panel slope in Shahrekord.

Fig 5a,5b, respectively, show the annual efficiency trends of the solar collector and the photovoltaic panel under different tilt angles. According to these two graphs, the tilt angle of the panel and collector varies from 17° to 47°. Fig 5a indicates that increasing the collector angle up to 24° results in the highest efficiency, which is 0.402. Likewise, the panel efficiency trend in Fig 5b shows that a tilt angle of 47° yields the maximum efficiency of 0.1842 compared to lower angles.

The seasonal variations are due to the high elevation of Shahrekord and the impact of mountainous topography on solar radiation patterns, which reduce direct radiation in winter and enhance it in summer. Studies conducted in similar cold and mountainous climates confirm this pattern. Researchers generally recognize that the optimal tilt angle depends on the latitude and the day of the year. Throughout the year, the tilt angle of the panel and collector is typically set equal to the latitude (ϕ); during the colder months, angles greater than the latitude (ϕ + 15) usually yield the best performance, while in summer, angles less than the latitude (ϕ – 15) are often more efficient [21].

### 3.2. Optimization of the tilt angle of the collector and solar panels in the Yazd pasteurization system

Yazd, located on the central plateau of Iran (31.9° N, 54.4° E) at an elevation of approximately 1230 m, has a hot and arid climate with very hot summers (average temperatures in August ranging from 35–40 °C) and mild winters (average temperatures in January from 5–10 °C). With an annual average solar irradiation of approximately 20,500 kJ/m^2^/day (equivalent to 5.69 kWh/m^2^/day), due to its clear skies and low humidity, Yazd is considered one of the most suitable regions for harnessing solar energy. This feature, along with Yazd’s distinct climate compared to Shahrekord (cold and mountainous) and Bandar-e-Abbas (hot and humid), made Yazd an ideal option for simulating the solar pasteurization system in this study.

Optimizing the tilt angle of flat plate collectors and photovoltaic panels for maximum solar radiation absorption enhances both thermal and electrical efficiencies, reducing reliance on auxiliary systems, such as electric heaters. The results of optimizing the collector and panel tilt angles in Yazd, using the RSM method and Design-Expert software, are presented in Table 3. The optimal collector angles ranged from 16.90° (June and July) to 46.90° (January, February, and December), while the panel angles varied between 16.90° (cold months) and 46.90° (hot months).

**Fig 6:**
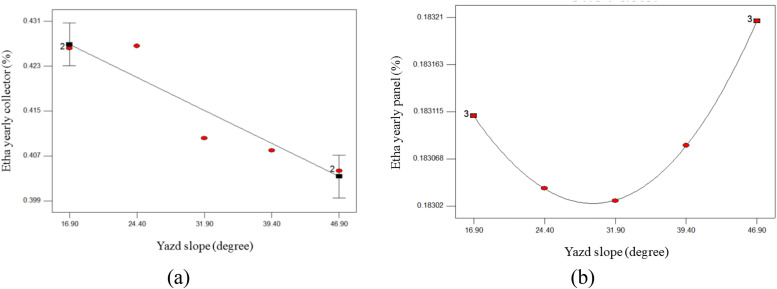
(a) Optimal collector slope, (b) optimal photovoltaic panel slope in Yazd.

The collector efficiency reached a maximum of 0.5685 in August, attributed to strong solar radiation and a lower tilt angle (17.14°). In contrast, in January, it dropped to a minimum of 0.3986 due to the relative decrease in winter solar radiation. The panel efficiency varied between 0.1817 (August) and 0.1865 (January), indicating the impact of high summer temperatures on reducing electrical efficiency. The annual efficiencies of the collector and panel were 0.4263 and 0.1832, respectively, at optimal angles of 17.24° and 46.90°, consistent with the one-factor plots from Design-Expert software, which demonstrated how efficiency varies with tilt angle (Fig 6a and b). Studies in similar arid climates support this pattern.

Verma et al., (2023) reported that a 43.5° angle for bifacial photovoltaic modules under hot and dry conditions resulted in a 24% efficiency [22], which aligns well with the 46.90° angle for the monofacial panels in Yazd (annual efficiency of 0.1832), although the lower efficiency here is related to the module type. In another study by Abdolzadeh et al. (2012), the optimization of solar collector tilt angles in hot and dry climates of Iran was investigated. Yazd was also included in that study, and the results closely matched the findings of the present research. For example, according to their study, the optimal annual tilt angles for January, February, March, and April were reported as 57°, 47.58°, 33.53°, and 21.53°, respectively, which are very close to the optimal angles obtained in this work [23].[22], which aligns well with the 46.90° angle for the monofacial panels in Yazd (annual efficiency of 0.1832), although the lower efficiency here is related to the module type. In another study by Abdolzadeh et al. (2012), the optimization of solar collector tilt angles in hot and dry climates of Iran was investigated. Yazd was also included in that study, and the results closely matched the findings of the present research. For example, according to their study, the optimal annual tilt angles for January, February, March, and April were reported as 57°, 47.58°, 33.53°, and 21.53°, respectively, which are very close to the optimal angles obtained in this work [23].

### 3.3. Optimization of the tilt angle of the collector and solar panels in the Bandar-e-Abbas pasteurization system

Bandar-e-Abbas (27.2° N, 56.3° E, at nearly sea level), located along the Persian Gulf, has a hot and humid climate with sultry summers (average August temperatures ranging from 32–38 °C, humidity 70–80%) and mild to warm winters (average January temperatures 18–22 °C). Its annual solar irradiation is approximately 19,800 kJ/m^2^/day (5.5 kWh/m^2^/day). Precisely adjusting the tilt angles of the collector and panels is crucial to overcoming challenges such as humidity, salt deposition, and corrosion, thereby ensuring system efficiency and minimizing the need for auxiliary energy.

**Fig 7:**
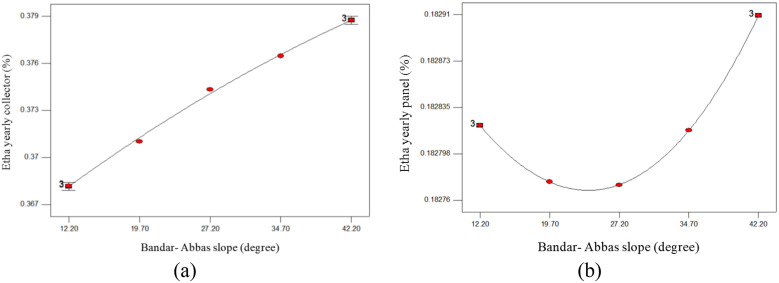
(a) Optimal collector slope, (b) optimal photovoltaic panel slope in Bandar-e-Abbas.

The tilt angle optimization using the response surface methodology (RSM) is presented in Table 4. The collector tilt angle varied from 12.20° (May–June) to 42.20° (January and November), while for the panels it ranged from 12.20° (cold months) to 42.20° (hot months). The collector efficiency reached 0.4209 in November at a tilt angle of 42.20°, due to strong solar radiation and lower humidity; however, it dropped to 0.3471 in February because of cloud cover. The panel efficiency was higher in winter (0.1851 in January) than in summer (0.1822 in June), attributed to milder temperatures. The annual optimal tilt angle was determined to be 42.20° for both the collector (yielding an efficiency of 0.4077) and the panel (yielding an efficiency of 0.1831). The data in the Table align with Fig 7(a), (b), which illustrate the impact of tilt angle variations on the annual efficiency of these systems.

**Table 4:** Monthly and annual optimization of tilt angles for solar panels and collectors in Bandar-e-Abbas using the RSM method.

Time	Collector Tilt	Collector Efficiency	Panel Tilt	Panel Efficiency
January	42.20	0.3764	12.20	0.1851
February	40.54	0.3470	12.20	0.1842
March	32.72	0.3518	12.20	0.1836
April	19.96	0.3776	42.20	0.1828
May	12.22	0.4071	42.20	0.1822
June	12.20	0.3856	42.20	0.1822
July	18.82	0.3911	42.20	0.1822
August	29.73	0.3899	42.20	0.1822
September	30.12	0.3978	42.20	0.1821
October	41.11	0.4061	12.20	0.1827
November	42.20	0.4209	12.20	0.1837
December	42.17	0.3694	12.20	0.1847
Yearly	42.20	0.4077	42.20	0.1831

Research on optimizing solar panels in coastal and humid climates highlights several key findings. For example, the optimal tilt angle for solar panels varies by location, and a linear relationship has been observed between the annual optimal tilt angle and latitude [24]. According to a study by Amani et al. (2021), in hot and humid climates, an optimal angle of 31° was found to maximize energy production [25]. Previous studies have also demonstrated that humidity significantly impacts the performance of solar panels, particularly in coastal areas, resulting in fluctuations in energy output as a function of temperature [26]. These findings underscore the importance of considering local climatic factors, particularly humidity, when designing and optimizing solar systems in coastal regions.[24]. According to a study by Amani et al. (2021), in hot and humid climates, an optimal angle of 31° was found to maximize energy production [25]. Previous studies have also demonstrated that humidity significantly impacts the performance of solar panels, particularly in coastal areas, resulting in fluctuations in energy output as a function of temperature [26]. These findings underscore the importance of considering local climatic factors, particularly humidity, when designing and optimizing solar systems in coastal regions.

### 3.4. Amount of total solar radiation

Based on the simulations performed and the data obtained from these simulations using computer software, the maximum radiation levels for each city were determined throughout the year. The total received radiation (I_T) on the collectors at the optimal tilt angles for each city was analyzed. The months with the highest radiation for Shahrekord, Yazd, and Bandar-e-Abbas were June, September, and April, respectively, corresponding to the distinct climates of each city. The maximum radiation values for Shahrekord, Yazd, and Bandar-e-Abbas were 750,000, 8,312.154, and 5,612.7 kJ/m^2^, respectively. Among these cities, Yazd receives the highest amount of solar radiation. According to the study by Asl et al. (2017), the highest solar radiation in central Iran is particularly observed in Yazd Province.

### 3.5. Solar water heating system

**Fig 8:**
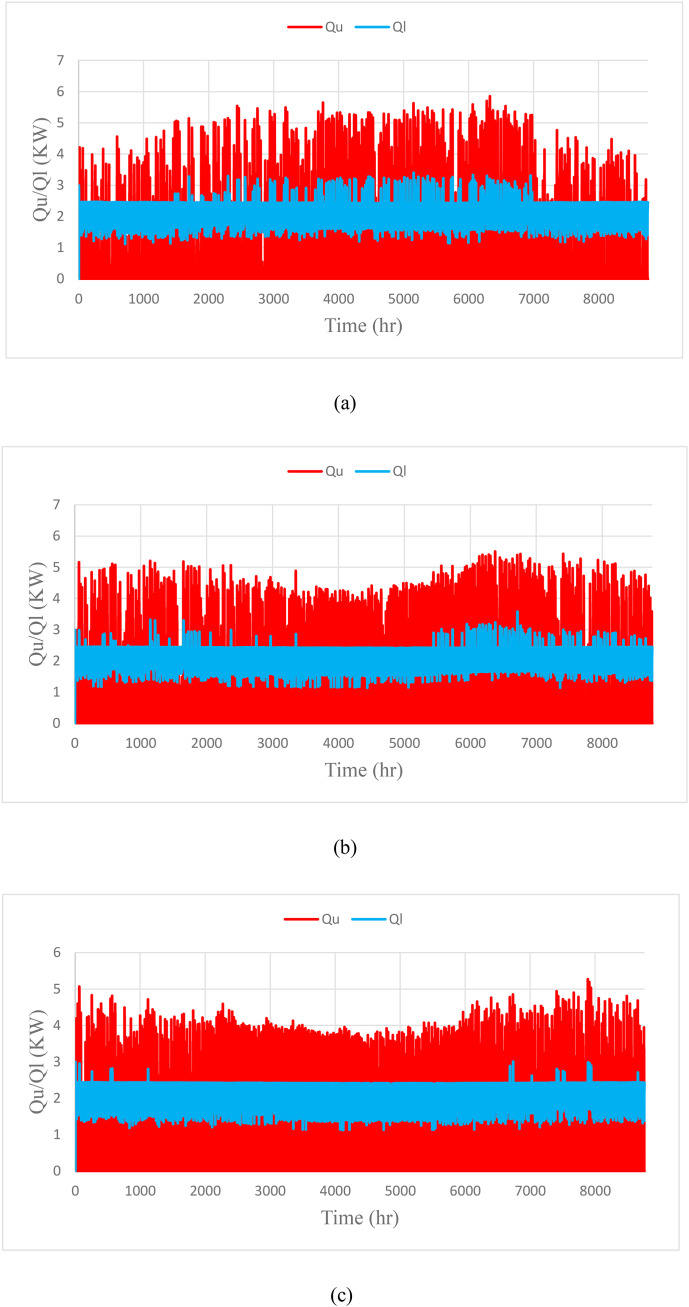
Useful energy gain by solar collector (Qu), the required energy for the pasteurization system (Ql) in (a) Shahrekord, (b) Yazd, and (c) Bandar-e-Abbas.

The thermal demand of the milk pasteurization system is primarily met by the solar water heating system, with a portion also supplied by the auxiliary system installed in the tank. There are three key parameters in meeting the thermal needs of the solar pasteurization system: the useful heat produced by the solar collector (Qu), the heat transferred from the solar collector to the pasteurization system (Ql), and the heat consumed by the auxiliary system to carry out the pasteurization process (Qaux). The results of this simulation, along with the data obtained for all three cities over a one-year period, are shown in Fig 8.

#### 3.5.1. Analysis of Useful Energy Gain (Qu) in solar collector performance.

According to these charts, the highest energy production by the solar collectors in Shahrekord occurs around mid-September (14th or 15th), reaching 5.85 kW. Similarly, the maximum useful energy produced in Yazd and Bandar-e-Abbas was recorded on September 16–17 and November 27–28, respectively, with values of 5.50 kW and 5.26 kW. These values and their timing depend on the climate of each city.

**Fig 9:**
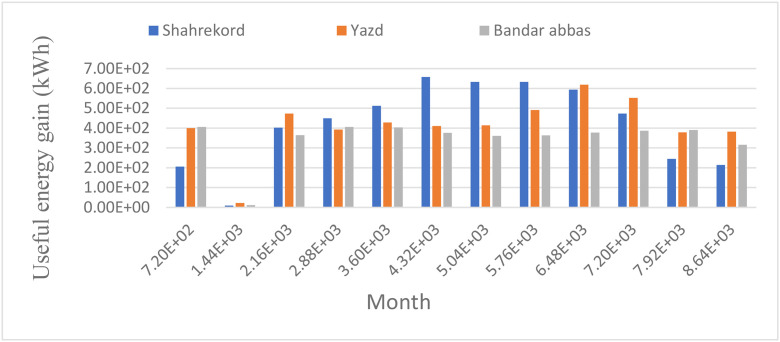
Useful energy gain in (a) Shahrekord, (b) Yazd, and (c) Bandar-e-Abbas.

Fig 9 presents a comparison of the heat generated by the solar system in the three cities of Shahrekord, Yazd, and Bandar-e-Abbas over the 12 months of the year. The data in this chart represents the amount of useful heat produced during one month, equivalent to 720 hours. In other words, these data reflect the average useful heat production under all conditions, including low sunlight hours (morning, evening, and night) and even cloudy days. This value differs from the hourly heat production reported for each city in the previous section.

Based on the above and the data from Fig 9, the useful heat produced in these three cities is compared, with Shahrekord having the highest amount, followed by Yazd, and finally the coastal city of Bandar-e-Abbas. According to this chart, the lowest useful heat production in all three cities occurred in the second month of the year, February, with values of 21.9 kWh for Yazd, 11.5 kWh for Bandar-e-Abbas, and 8.8 kWh for Shahrekord, respectively. Although solar altitude is generally lower in January, the simulation results revealed that February exhibited the lowest useful energy gain (Qu) and energy delivered to the load (Ql) across all three cities. This unexpected pattern can be attributed to less favorable climatic conditions in February, including higher cloud cover, increased atmospheric humidity, and more frequent precipitation events compared to January. These factors led to a notable reduction in both direct and global solar radiation, which outweighed the slight improvement in solar altitude. Additionally, the lower ambient temperatures in February increased thermal losses from the collector and storage tank, further decreasing overall system performance [27].[27].

This observation highlights the importance of using actual hourly meteorological data rather than relying solely on solar geometry when evaluating solar system performance in different seasons.

According to this chart, among the three cities, Shahrekord produces the highest amount of useful heat from the system, reaching 657 kWh in June. Overall, based on this chart, it can be concluded that Shahrekord has the greatest useful heat production from early spring (April) through late summer (September) compared to the other two cities. Due to its high altitude, Shahrekord experiences less atmospheric scattering, resulting in stronger direct solar radiation in spring and summer compared to Yazd and Bandar-e-Abbas. Therefore, it can be concluded that the performance of the solar pasteurization system in Shahrekord during spring and summer is better than in the hot and dry climate of Yazd and the humid coastal climate of Bandar-e-Abbas.

Additionally, the data from this chart indicates that Yazd produces more useful heat than the other two cities during most of the cold months, such as February, March, and even September and October, as well as December. Comparing the climates of these three cities, it can be argued that Yazd, due to its warm, dry, and desert climate, clear skies, high atmospheric transparency, moderate temperatures in the cold months, and strong direct solar radiation, generates more useful solar energy compared to Shahrekord (which likely experiences more cloudiness and colder temperatures) and Bandar-e-Abbas (which has higher humidity and more diffuse radiation).

By further examining this chart, it can be observed that Bandar-e-Abbas produces the lowest amount of useful heat compared to the other two cities for most months of the year, except for January and November, during which it slightly surpasses Yazd to rank first among the three. In these two months, Bandar-e-Abbas typically experiences relatively favorable weather conditions, with clearer skies and lower humidity compared to other times of the year. This increases atmospheric transparency and allows for more direct solar radiation, which is crucial for generating useful heat.

Overall, due to its coastal climate and high humidity, Bandar-e-Abbas experiences reduced efficiency of solar panels and thermal collectors during the hot months, as very high temperatures and intense humidity lower the panel voltage output and cause dust and pollution to accumulate on the panel surfaces. Yazd, on the other hand, enjoys mostly clear and bright skies throughout the year. The slight difference in solar incidence angles between November and January in Yazd compared to Bandar-e-Abbas likely results in somewhat lower useful heat production in Yazd than in Bandar-e-Abbas during those months.

Iran, with more than 300 sunny days per year and an average annual solar irradiation of about 2300 kWh/m^2^, possesses significant potential for solar energy [28]. Remarkably, this figure exceeds the global average, as Iran is situated within the world’s solar belt [29]. Studies estimate that utilizing just 1% of Iran’s total land area for solar energy could generate approximately 9 million MWh per day [28].[28]. Remarkably, this figure exceeds the global average, as Iran is situated within the world’s solar belt [29]. Studies estimate that utilizing just 1% of Iran’s total land area for solar energy could generate approximately 9 million MWh per day [28].

#### 3.5.2. Analysis of energy to load (Ql) in solar collector performance.

Ql is one of the most important parameters in evaluating solar systems because it determines how much of the produced thermal energy is delivered to the load. The load can be any device or equipment used within the system under consideration. In this study, the load refers to the milk pasteurization system. Determining the value of Ql for all three cities enables a more comprehensive analysis and comparison of the solar system’s performance under varying climatic conditions.

**Fig 10:**
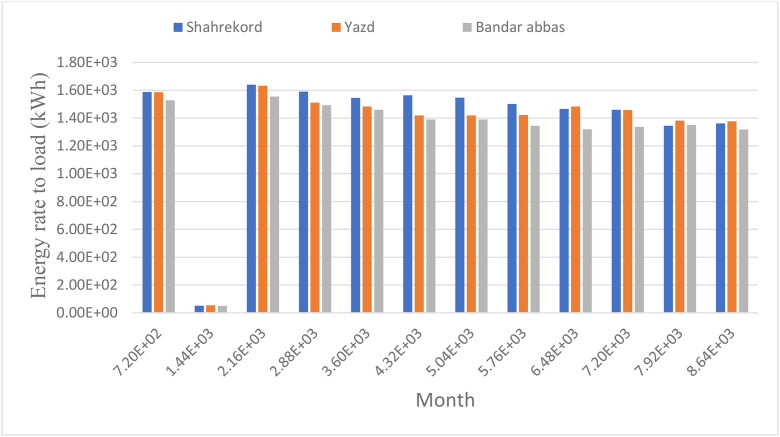
Energy rate to load (Ql) in (a) Shahrekord, (b) Yazd, and (c) Bandar-e-Abbas.

Fig 10 presents a monthly comparison for the cities of Shahrekord, Yazd, and Bandar-e-Abbas, illustrating the amount of useful heat produced by each city that was transferred to the pasteurization system throughout the year. According to this chart, Ql in all three cities ranged from approximately 50 kWh to about 1640 kWh. Among all the months, February transferred the least amount of heat to the pasteurization system in all three cities, which aligns with the previous section where the lowest useful heat production was also reported in this month. The chart also indicates that the third month of the year, March, showed the best performance for all three cities. The highest Ql values in Yazd and Bandar-e-Abbas were recorded at 1630 kWh and 1560 kWh, respectively.

With a more detailed and precise analysis of this section, it can be concluded that overall, Shahrekord performed better than Yazd and Bandar-e-Abbas in most months of the year. According to the available data, Shahrekord was able to transfer up to 1640 kWh of the useful heat produced to the pasteurization system, which is the highest amount of energy delivered to the load compared to the other two cities.

As mentioned earlier, Shahrekord demonstrated significantly better performance than the other two cities during the spring and summer, while Yazd, with only a slight difference, ranked second in these seasons. A closer look at the system’s performance during the colder months reveals that the pasteurization system in Yazd can actually achieve better efficiency than That in Shahrekord during cold periods, placing it in the top position. This is mainly due to Yazd’s clear and calm skies during the colder months. Overall, however, it can be concluded that Bandar-e-Abbas had a weaker performance than the other two cities across all seasons. This finding is consistent with the results reported by Dobrowsky et al., (2015), whose study also indicated that in humid climates, due to high moisture levels, the amount of energy transferred to the load is lower, and consequently, the efficiency of solar systems in these regions tends to be reduced compared to drier climates [30].[30].

#### 3.5.3. Analysis of energy provided by the auxiliary system (Qaux) in solar collector performance.

Auxiliary heating (Qaux) serves as a complement to the solar collector in the pasteurization system, designed to compensate for thermal energy shortages under low solar irradiation or unfavorable ambient temperatures. This section analyzes the monthly trends of Qaux in Shahrekord, Yazd, and Bandar-e-Abbas to assess the system’s dependency on auxiliary heating across different climates.

**Fig 11:**
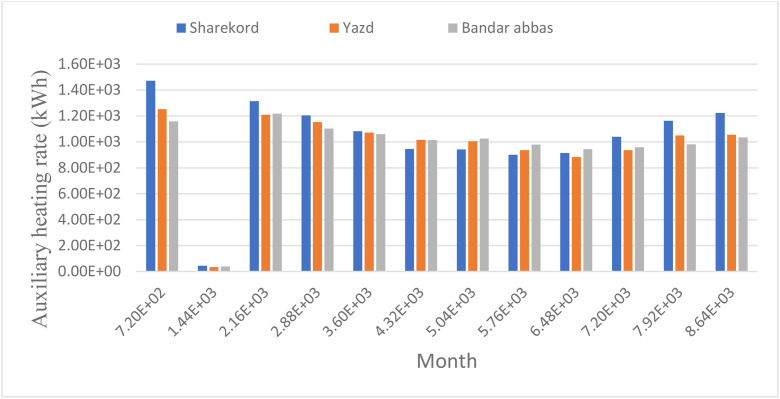
The energy provided by the auxiliary system (Qaux) in (a) Shahrekord, (b) Yazd and (c) Bandar-e-Abbas.

The bar charts (Fig 11) illustrate the impact of climatic factors on the auxiliary system’s operation. According to these charts, all three cities recorded the lowest use of the auxiliary system in February, which aligns with earlier discussions indicating that both useful energy production and energy delivered to the load were at their minimum during this month.

The data show that Shahrekord and Yazd had the highest demand for auxiliary heating in January, with 1,470 and 1,250 kWh supplied through the auxiliary system, respectively. Meanwhile, Bandar-e-Abbas reached its peak auxiliary demand in March with 1,220 kWh. The greater need for auxiliary heating in January in Shahrekord and Yazd is attributed to reduced solar irradiation during this time of year, consistent with findings by [31]. In their study, Atia and colleagues utilized a solar system for milk pasteurization. They demonstrated that solar systems are economically viable for high-consumption processes, such as pasteurization, achieving 7.5 liters of pasteurized milk per hour. However, they also noted increased reliance on the auxiliary system during periods of low solar irradiation.[31]. In their study, Atia and colleagues utilized a solar system for milk pasteurization. They demonstrated that solar systems are economically viable for high-consumption processes, such as pasteurization, achieving 7.5 liters of pasteurized milk per hour. However, they also noted increased reliance on the auxiliary system during periods of low solar irradiation.

Overall, the chart for Shahrekord reveals that from late spring through late winter, the system required relatively low support from auxiliary heating, indicating that the solar system effectively met the thermal demands. From June to September, auxiliary usage ranged between 900 and 944 kWh, with August showing the lowest value at 900 kWh. This aligns with previous data indicating high useful heat production and energy delivered to the load in Shahrekord during August, resulting in less reliance on the auxiliary system.

Conversely, Yazd required minimal auxiliary heating during autumn and the colder months, primarily due to its hot and dry climate, ample solar irradiation, and clear skies compared to Shahrekord. The auxiliary system in Yazd supplied 937, 883, and 937 kWh in September, October, and December, respectively.

Bandar-e-Abbas, with its humid coastal climate, exhibited a pattern similar to Yazd, with significantly reduced auxiliary heating needs during autumn and winter. This suggests that, among the three cities with distinct climates, Bandar-e-Abbas performed relatively better during colder months, reducing dependence on auxiliary heating to an acceptable level. Data indicate that from August to November, auxiliary usage ranged from 943 to 981 kWh.

In general, the overall trend suggests that Shahrekord maintained a well-balanced demand between cold and warm months, resulting in an energy profile that appears both reasonable and efficient. This likely positions Shahrekord as a more favorable candidate for implementing this type of solar system.

### 3.6. Photovoltaic panel performance

In this study, the most important component for supplying electrical energy to the solar pasteurization system is the photovoltaic panels. The electrical energy supply for the system includes powering the combined Venturi tube and liquid-phase plasma pasteurization system, as well as the electric heater used as an auxiliary system and warm load. After simulating and collecting data over one year for the three cities of Shahrekord, Yazd, and Bandar-e-Abbas, the maximum and minimum power outputs of the photovoltaic panels used in this study for each city were determined and are reported in Table 5.

According to this Table, the highest power output was recorded in Yazd and Shahrekord (712 kWh). Reviewing findings from previous studies highlights the importance of considering local climatic conditions when implementing solar energy systems in Iran to maximize energy production and ensure economic feasibility. A multi-criteria assessment identified Yazd as the optimal location for photovoltaic systems, offering the highest energy yield and efficiency [32]. In that study, a solar system was installed and compared across various Iranian cities with different climatic conditions. Yazd and Bandar-e-Abbas were among the cities investigated, and the results showed that photovoltaic panels achieved the highest power output and best performance in Yazd, followed by Isfahan and then Bandar-e-Abbas, which is consistent with the findings of the present study.[32]. In that study, a solar system was installed and compared across various Iranian cities with different climatic conditions. Yazd and Bandar-e-Abbas were among the cities investigated, and the results showed that photovoltaic panels achieved the highest power output and best performance in Yazd, followed by Isfahan and then Bandar-e-Abbas, which is consistent with the findings of the present study.

### 3.7. Analysis of thermal storage tank performance

**Fig 12:**
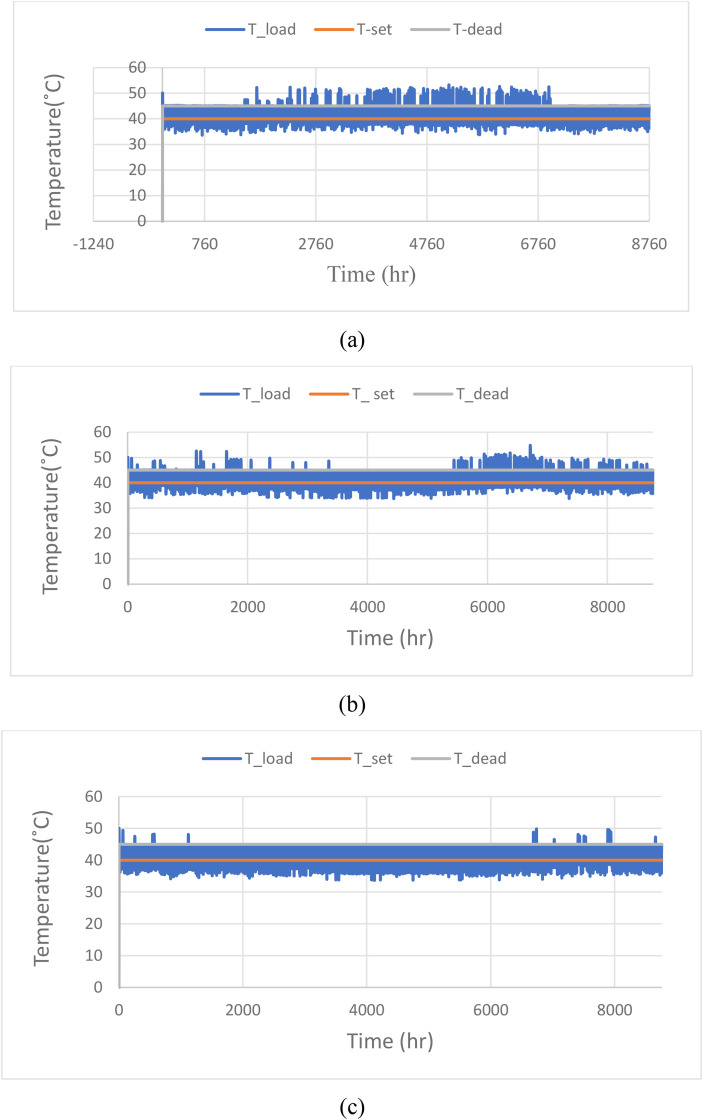
Tank temperature changes (T_load), set temperature (T_setpoint), and deadband temperature (T_deadband) in (a) Shahrekord, (b) Yazd, and (c) Bandar-e-Abbas.

The Analysis of tank performance in this study focuses on three key parameters: the tank temperature (T_load), the target temperature, and the permissible temperature band, all of which are illustrated in Fig 12.

The tank temperature represents the actual temperature of the fluid inside the tank. The target temperature denotes the desired or standard temperature of the system, which in this study was selected based on the optimal condition for milk pasteurization. According to the study by Taki et al. (2023), this target temperature was set at 40°C. Taki and colleagues demonstrated that milk pasteurization using a Venturi tube reactor combined with a liquid-phase plasma system, at a system temperature of 40.10°C, achieved a 5.42-log reduction of *Escherichia coli*. Therefore, this temperature was adopted as the target temperature for ensuring milk safety and quality in the current research.

Additionally, the dead band (ΔT) indicates the acceptable range of temperature fluctuations within the system. Fig 12 presents the tank performance across the three cities: Shahrekord, Yazd, and Bandar-e-Abbas. As depicted, the hourly tank temperature was recorded throughout the year, with a target temperature of 40°C and an allowable deviation of ±5°C, indicating an acceptable range of 35°C to 45°C.

Comparing these figures reveals that Bandar-e-Abbas exhibited a more stable and consistent temperature trend than Shahrekord and Yazd, whose graphs displayed more frequent fluctuations. For a more precise comparison, the mean and standard deviation of the tank temperature (T_load), critical for evaluating the temperature control system and thermal stability of the pasteurization system, are provided in Table 6.

**Table 6:** Average and standard deviation of tank temperature.

Cities	Average	Standard deviation
Shahrekord	42.9	3.246
Yazd	42.5	3.107
Bandar-e-Abbas	41.7	3.109

The mean tank temperature indicates how closely Shahrekord, Yazd, and Bandar-e-Abbas approached the 40°C target, confirming the system’s efficiency under different climatic conditions. The standard deviation reflects temperature variability; lower values (such as in Yazd) suggest greater thermal stability and reduced dependency on auxiliary heating (Qaux), whereas higher values (such as in Shahrekord) indicate climatic variability. These parameters facilitate tank design optimization and reduce energy consumption.

Among the three cities, Bandar-e-Abbas showed a slightly lower mean tank temperature (41.7°C), while Yazd had the lowest standard deviation at 3.107°C. The lower mean in Bandar-e-Abbas is attributed to its high humidity and lower Qu (5.26 kW), yet it still falls within the acceptable range of 35–45°C. Meanwhile, Yazd’s stable solar radiation and hot, dry climate resulted in less fluctuation in T_load (3.107°C), even though its mean temperature was higher than Bandar-e-Abbas (42.5°C).

Although a lower standard deviation is generally preferable, considering the primary objective of this study milk pasteurization the process is not highly sensitive to temperature, and fluctuations within approximately 5°C are acceptable. Therefore, the observed standard deviations across the three cities, ranging from 3.1 to 3.2°C, are within appropriate limits.

A study by Tigabe et al. (2022) examining the performance of a milk pasteurization system using a flat-plate collector in the hot climate of Ethiopia (ambient temperatures ranging from 14.3 to 29.7°C, similar to Bandar-e-Abbas and Yazd), reported that hybrid pasteurization systems required lower operational temperatures (40–50°C) than traditional methods [33]. This aligns well with the T_load of 40°C employed in the present study. Moreover, the reported standard deviation of tank temperatures (~2–4°C) in that study indicates sufficient thermal stability for milk pasteurization using flat-plate collectors, even under fluctuating solar irradiation conditions.[33]. This aligns well with the T_load of 40°C employed in the present study. Moreover, the reported standard deviation of tank temperatures (~2–4°C) in that study indicates sufficient thermal stability for milk pasteurization using flat-plate collectors, even under fluctuating solar irradiation conditions.

### 3.8. Supplying the heating and cooling load needs of the pasteurization system

**Fig 13:**
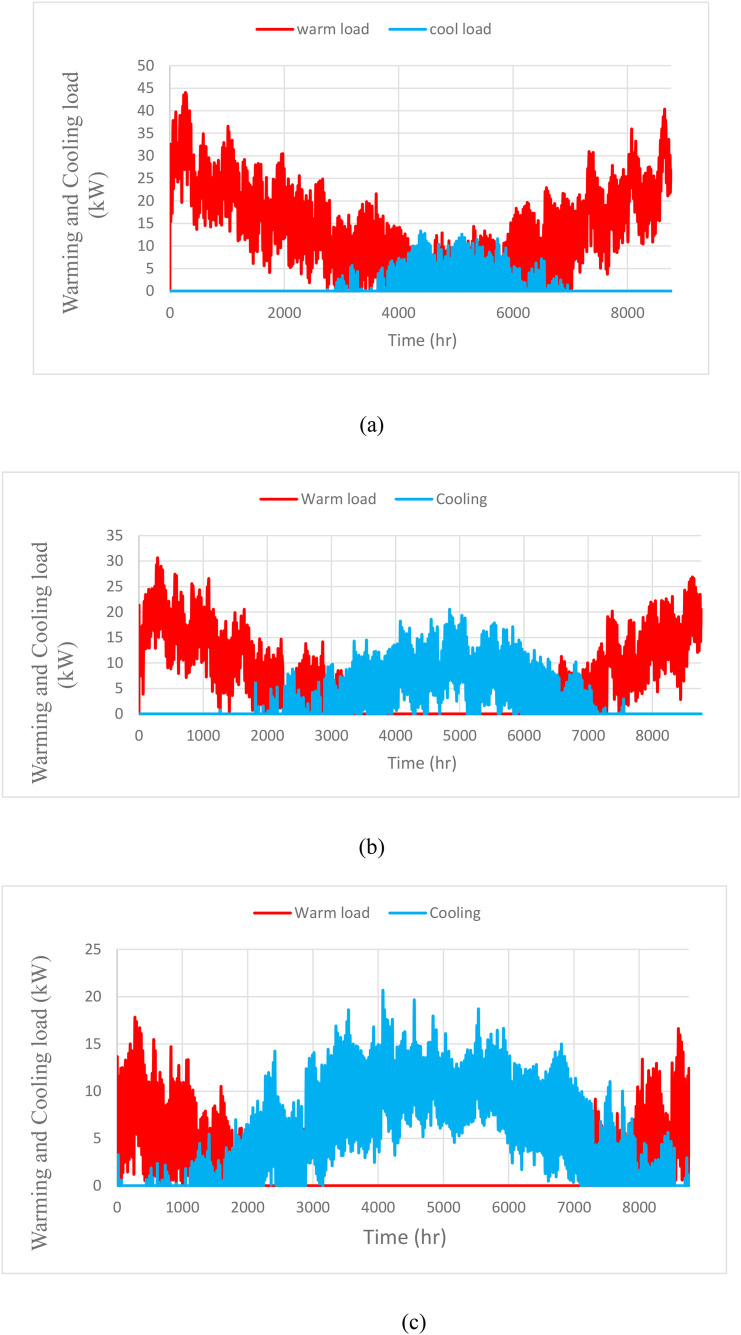
Warming and cooling requirements of the pasteurization system in (a) Shahrekord, (b) Yazd, and (c) Bandar-e-Abbas.

The provision of heating and cooling loads in solar-assisted systems varies significantly across different climates, influenced by factors such as ambient temperature, humidity, and solar irradiance [34]. [34]. Analyzing the heating demand (Qaux) and the cooling requirements of the critical zone in the solar pasteurization workshop, aimed at maintaining a temperature of 24°C, is essential for assessing system performance. This subsection evaluates the impact of the climates in Shahrekord, Yazd, and Bandar-e-Abbas on heating and cooling demands as well as on the temperature stability of the controlled workshop. Fig 13 illustrate these analyses, which were conducted dynamically over an entire year (8,760 hours).

According to previous studies, dynamic simulations demonstrate that solar-assisted heating and cooling systems can effectively balance energy demand, which is particularly crucial in Iran, where peak electricity demand is a major concern [35].[35].

From these figures, it is clearly evident that the cooling demand in Yazd and Bandar-e-Abbas is substantially higher than the heating demand of these two cities. Additionally, as illustrated in the charts, Shahrekord, with its mountainous and cold climate, experiences a higher heating load during the colder months. The peak heating load recorded was 44.10 kW in January, around January 12, which is consistent with the harsh, cold, and unstable climate of Shahrekord during this period.

The higher water level in the diagram for the city of Bandar-e-Abbas indicates that the cooling need in this city is significantly higher than the heating need. The higher blue level in the diagram for Bandar-e-Abbas shows that the cooling demand in this city is significantly higher than the heating demand. The peak cooling load in this city seems to occur during the month of June, which corresponds to the middle of the year, reaching values close to 70.20 kW. This sharp increase is consistent with the hot and humid climate of Bandar-e-Abbas (temperatures usually between 30–35°C and high solar radiation). The heating demand in this city is also low, with the highest demand occurring at around 80.17 kW, which typically happens in January.

The Yazd graph shows a lower cooling demand than Bandar-e-Abbas, but the cooling load is still significant during the warm months of the year (around 4000–5000 hours), with peaks around 20–21 kW. This reduction in cooling demand is consistent with Yazd’s hot and dry climate, where summer temperatures are high but humidity is lower than Bandar-e-Abbas. The heating load in Yazd is also very low and occurs mainly early in the year, consistent with the region’s mild to cold winter temperatures.

For a more comprehensive understanding and assessment of the heating and cooling system performance, it is crucial to examine the annual mean and standard deviation of the data, which are summarized in Table 7.

**Table 7:** Average and standard deviation of the warm load and cooling system.

Cities	Average of warm load	Standard deviation of warm load	Average of cooling	Standard deviation of cooling
Shahre-kord	13.40	10.06	0.726	2.10
Yazd	6.53	7.58	2.89	4.54
Bandar-e-Abbas	1.82	3.43	5.06	4.96

According to this Table, the mean and standard deviation of the heating (Qaux) and cooling demands of the workshop (24°C) illustrate the temperature control performance across Shahrekord, Yazd, and Bandar-e-Abbas. In Bandar-e-Abbas, the average heating demand was 1.82 kW, while the cooling demand was relatively high at 5.06 kW with a standard deviation of 4.96. This is attributable to its hot ambient temperatures (~30–35°C) and high annual solar irradiation (7,506,012 kJ/m^2^). Similarly, Yazd exhibited more heating requirements (6.53 kW) compared to Bandar-e-Abbas and significant cooling needs (2.89 kW).

These findings are consistent with the results of Allouhi et al. (2022), [36], who reported that hot and arid climates have negligible heating requirements, with systems predominantly requiring cooling throughout the year.[36], who reported that hot and arid climates have negligible heating requirements, with systems predominantly requiring cooling throughout the year.

Shahrekord exhibited a heating load of 13.40 kW and a cooling load of 0.726 kW, indicating greater fluctuations, which is consistent with the local climate characteristics. These values reveal that, unlike Yazd and Bandar-e-Abbas, Shahrekord with its generally lower ambient temperatures requires notable heating. Additionally, due to the prevalence of cold days, its cooling demand is considerably lower compared to Yazd and Bandar-e-Abbas.

Overall, based on previous studies, it can be stated that while space heating requirements exist across various climates, these demands tend to approach negligible levels. In cold and temperate-humid regions, heating needs are significantly higher, whereas hot, dry, and even humid climates demand substantially less heating energy [37].[37].

### 3.9. Solar fraction

The solar fraction (SF) represents the ratio of useful energy supplied by the system, which includes both solar and electrical contributions to the total energy demand of the system, encompassing auxiliary heating, cooling, and other loads. This parameter, recognized as one of the most critical indicators for evaluating solar system performance, effectively quantifies the extent to which the system’s energy requirements are met by solar energy. In essence, it serves as a direct metric of the solar system’s utility.

A higher solar fraction indicates reduced dependence on fossil fuels and electricity, which translates into economic advantages. However, it is important to note that a higher SF is not always desirable, as it may also reflect overproduction of energy and suboptimal utilization of the installed equipment. In such cases, integrating energy storage solutions becomes essential to capture and utilize the excess energy generated.

Previous studies have demonstrated that the solar fraction is a key parameter in solar thermal power plants, typically ranging between 5% and 60% in industrial processes. Moreover, optimized parabolic collector networks have been reported to potentially achieve an average annual solar fraction of up to 1.05, highlighting the considerable promise of well-designed systems [38].[38].

**Fig 14:**
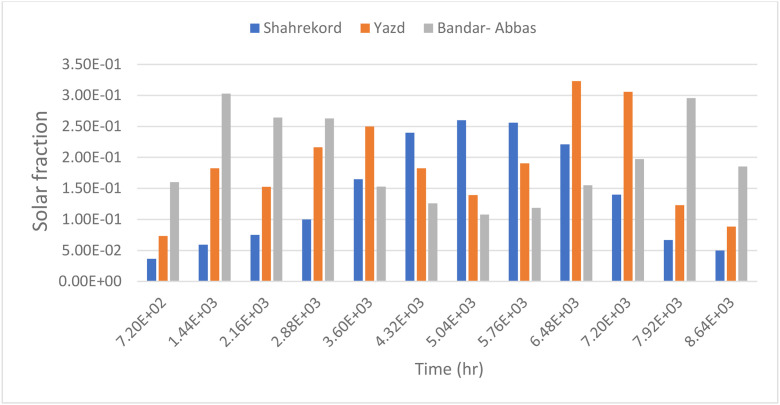
Solar fraction in (a) Shahrekord, (b) Yazd, and (c) Bandar-e-Abbas.

In this study, the solar fraction (SF) was calculated for all three cities, and the monthly results were presented in a bar chart shown in Fig 14. According to this figure, the maximum solar fraction among these cities was observed in Yazd, reaching 0.323 in September, due to its high solar radiation (8,312,154 kJ/m^2^) and milder temperatures (15–25°C) during this month of the year, which enhance PV efficiency. Bandar-e-Abbas also demonstrated a notable maximum solar fraction of 0.30 in February, ranking second after Yazd. The figure for Shahrekord also shows a solar fraction of 0.26 in July.

A review of previous studies indicates that the solar fraction tends to decrease with an increase in the thermal load demanded by the solar system and the collector area. This effect is more pronounced in winter due to reduced solar irradiation [39,40].

Overall, the range of solar fraction variations for each city was as follows: Shahrekord (0.036–0.26), Yazd (0.07–0.32), and Bandar-e-Abbas (0.10–0.30). The solar-assisted design, prioritizing microbial inactivation over maximum solar utilization, results in moderate SF values compared to fully solar systems (0.57 in [41]).

A study conducted in Shahrekord aimed to meet the space heating, domestic hot water, and swimming pool demands of a residential building. It reported a solar fraction of approximately 41% for the solar systems, showing close agreement with the present findings for Shahrekord [42].[42].

In another study, the annual solar fraction for solar systems was measured across three different climatic zones in Iran. According to this study, the annual SF was found to be 0.57 in the semi-arid regions of the central plateau, 0.24 in the humid southern coastal regions, and 0.51 in cold mountainous areas [41]. The solar fraction obtained in this study (0.036–0.32) is lower than the value of 0.57 reported in some previous Iranian studies [36]. This difference is primarily due to the smaller capacity of the system (600 l/day for a 30-cow farm), the low operating temperature (40 ± 5°C) required for the non-thermal Venturi-plasma pasteurization (compared to conventional thermal processes), and the additional electrical load of the plasma reactor supplied by the PV system. These design choices prioritize quality preservation and suitability for remote small-scale farms over maximizing solar fraction.[41]. The solar fraction obtained in this study (0.036–0.32) is lower than the value of 0.57 reported in some previous Iranian studies [36]. This difference is primarily due to the smaller capacity of the system (600 l/day for a 30-cow farm), the low operating temperature (40 ± 5°C) required for the non-thermal Venturi-plasma pasteurization (compared to conventional thermal processes), and the additional electrical load of the plasma reactor supplied by the PV system. These design choices prioritize quality preservation and suitability for remote small-scale farms over maximizing solar fraction.

In addition to the technical performance indicators, previous techno-economic investigations on hybrid photovoltaic systems have demonstrated that optimal sizing of PV arrays can significantly reduce the levelized cost of energy (LCOE) and improve the economic feasibility of renewable energy systems in remote regions. Such studies indicate that integrating solar thermal and photovoltaic subsystems with optimized operational strategies can reduce long-term operational costs while improving energy reliability. Therefore, although a detailed economic analysis was beyond the scope of the present study, the obtained solar fractions and stable thermal performance suggest that the proposed solar-assisted Venturi-plasma pasteurization system has strong potential for cost-effective operation in decentralized dairy farms and energy-constrained rural areas.

### 3.10. Effectiveness analysis and outlook

The combined Venturi tube and liquid-phase plasma system leverages hydrodynamic cavitation and reactive species to achieve microbial inactivation in milk. As validated by Taki et al. (2023), this system, powered by grid electricity, achieved over 5 log reduction of *Escherichia coli*, extending milk shelf life by 20–30 days without compromising sensory attributes (color difference ΔE = 0.41, taste score 6.5 compared to 7.2 for control) or nutritional properties (e.g., proteins, vitamins). The synergistic effect arises from cavitation-induced shock waves and bubble collapse, which physically disrupt microbial cells, and plasma-generated reactive oxygen and nitrogen species (hydroxyl radicals, ozone), which chemically damage cell membranes and proteins [18]. The Venturi tube enhances turbulence (turbulent kinetic energy, TKE) and bubble formation, amplifying plasma efficacy by improving mass transfer of reactive species across liquid layers.[18]. The Venturi tube enhances turbulence (turbulent kinetic energy, TKE) and bubble formation, amplifying plasma efficacy by improving mass transfer of reactive species across liquid layers.

The current study extends this work by simulating the same Venturi-plasma system with solar preheating for a 600 l/day pasteurization process (T_load = 40 ± 5°C). The shift to solar energy does not alter the microbial inactivation mechanism, as the Venturi-plasma chemistry remains unchanged, but focuses on utilizing clean energy.

The dynamic yearly simulation with optimized collector and panel tilt angles was specifically conducted to achieve stable system performance and minimize the impact of solar intermittency. The results demonstrate that the storage tank successfully maintained the target temperature (40 ± 5°C) for the majority of operating hours across all three climates. Although transient fluctuations in solar radiation may occur in real conditions, the thermal inertia of the storage tank and the control strategy effectively buffer these variations, ensuring consistent preheating conditions for the Venturi-plasma unit.

Future work could explore scaling the system beyond 1000 l/day to assess its viability for industrial settings, integrating energy storage systems to reduce auxiliary energy (Qaux), and conducting experimental validations to confirm microbial and quality outcomes under solar-powered conditions. Employing advanced solar collectors could also enhance performance in humid climates like Bandar-e-Abbas, where high humidity (50–70%) results in lower SF. These advancements would enhance the system’s potential as a sustainable and energy-efficient alternative for dairy processing.

### 3.11. Experimental validation of the pasteurization module and overall study implications

The present simulation study is built upon a robust experimentally validated technology. In previous experimental investigations using the identical Venturi tube hydrodynamic cavitation and liquid-phase cold plasma reactor [3,43], the system successfully achieved a 5.42 log reduction of *Escherichia coli* in milk at the target temperature of 40.10 ± 5 °C. This inactivation level satisfies the microbiological criteria for effective milk pasteurization. Moreover, the non-thermal treatment demonstrated excellent quality preservation, with no statistically significant adverse effects on key physicochemical properties including pH, fat, protein and lactose content, color (ΔE < 1.0), and oxidative stability (low TBARS values). These results confirm the synergistic efficacy of hydrodynamic cavitation which enhances turbulence, mass transfer, and mechanical cell disruption and liquid-phase plasma-generated reactive species for microbial inactivation, while effectively minimizing nutrient degradation and sensory alterations commonly associated with thermal pasteurization.

By integrating this experimentally proven non-thermal pasteurization module with solar thermal collectors and photovoltaic panels, the current study demonstrates the technical feasibility and climatic adaptability of a renewable-energy-driven system for small-scale dairy processing. The achieved solar fractions, collector efficiencies, and stable temperature control across diverse Iranian climates collectively indicate strong potential for sustainable and decentralized milk pasteurization, particularly in remote and energy-constrained regions.

## 4. Conclusion

This study introduced a pioneering solar-powered milk pasteurization system combining a Venturi tube reactor with liquid-phase cold plasma. Simulations across three distinct climates in Iran demonstrated the system’s robustness, with annual efficiencies reaching up to 0.47 for collectors and 0.18 for PV panels. Yazd recorded the peak solar radiation (8311 kWh/m²), while Shahrekord offered superior performance across most months, confirming the system’s adaptability. The achieved solar fraction (0.036–0.32) underscores a significant reduction in fossil fuel reliance, aligning with sustainability goals. The achieved solar fraction (0.036–0.32) is reasonable for a small-scale solar-assisted pasteurization system (600 l/day) across different Iranian climates. However, the system still shows considerable reliance on auxiliary heating during low solar radiation periods. Future studies could improve the solar fraction by increasing the collector area or storage tank capacity through sensitivity analysis and techno-economic optimization. These findings are generalizable to other high-altitude, arid, and humid coastal regions globally. While the system shows great potential for eco-friendly milk pasteurization in remote areas, future studies should focus on integrating energy storage solutions to further minimize auxiliary heating demands and enhance operational cost-effectiveness under varying solar conditions, and also, a detailed life-cycle assessment (LCA) is recommended for future studies to accurately quantify the CO_2_ emission savings associated with the displaced grid electricity. While a comprehensive economic analysis was not performed in this study, the technical results suggest strong potential for reducing fossil fuel dependency in remote dairy farms. Future research should include detailed techno-economic evaluation to fully assess the cost-effectiveness of the proposed system.

## Supporting information

S1 Table Collecting data. (XLSX)
