# Supplementary material for: Simulation and performance analysis of a solar-assisted venturi-plasma pasteurization system for milk
Source: PLoS One. 2026 Jul 2;21(7):e0352384. doi: 10.1371/journal.pone.0352384 (PMC13327238; doi:10.1371/journal.pone.0352384)
Supplement: Attachment — Submitted filename: answer to reviewer.docx [file pone.0352384.s003.docx]

Response to the honorable referee of the paper entitled:

**Simulation and Performance Analysis of a Solar-Assisted Venturi-Plasma Pasteurization System for Milk**

Thanking the comments and proposed amendments of the honorable referees, the answer to the referred points is mentioned in the separation of different parts of the paper as follows:

Reviewer #1:

1. While the introduction identifies that previous studies focused on conventional thermal methods, it should more clearly articulate why a non-thermal "Venturi-plasma" system specifically requires solar assistance compared to other technologies.

Response: **Thanks for this constructive comment.** In response to the reviewer’s comment, in introduction section we emphasize that although the Venturi–plasma pasteurization system is principally non‑thermal, a mild solar-assisted preheating (up to 35–40 °C) significantly enhances hydrodynamic performance and microbial reduction without compromising the non‑thermal nature. Moreover, photovoltaic panels supply the required electrical energy for plasma generation and control units through renewable sources, enabling fully off‑grid operation. This integration allows feasible deployment in remote dairy farms where conventional energy supply is limited, thus underscoring the necessity and novelty of solar assistance in this system.

2. The text briefly mentions "liquid-phase cold plasma" and "hydrodynamic cavitation". A more detailed explanation of why these specific non-thermal methods were chosen over other emerging technologies (e.g., Pulsed Electric Fields or UV-C) would strengthen the rationale.

Response: thanks for your benefit comment. We chose the Venturi-plasma system because it is a non-thermal method with a strong synergy between its components. In the Venturi tube, pressure drops and velocity changes create a high turbulence flow. This turbulent environment helps to inactivate microorganisms and creates cavitation, which weakens the microbial cell walls. By combining this with liquid-phase plasma which is an innovative approach the plasma is generated directly inside the milk. While the plasma provides disinfection, the turbulence from the Venturi helps to distribute the generated radicals throughout the liquid. This makes the treatment more uniform and effective than UV-C (which cannot penetrate milk) or PEF (which faces fouling issues).

3. The introduction mentions high economic costs of traditional systems but does not provide a baseline for current energy costs in Iran to better frame the "cost-effective" claim made later.

Response: Thank you for this valuable comment. In this study, our primary focus was on the technical feasibility and energy performance of the proposed solar-assisted system. The term “cost-effective” was mainly used in the context of remote and off-grid dairy farms (equivalent to a 30-cow farm), where access to reliable grid electricity is limited or nonexistent. In such areas, any reduction in dependence on fossil fuels or diesel generators can significantly lower operational costs. However, we acknowledge that a detailed economic analysis (payback period or life-cycle cost) was not performed in the current work. We have revised the manuscript by moderating the economic claims and adding a clarifying sentence in the Introduction and Conclusion to better reflect the scope of the study.

4. The paper mentions "computer software" several times for simulation (e.g., in sections 2.1 and 2.6). The specific software name (e.g., TRNSYS, MATLAB, or a custom build) should be explicitly stated in the Materials and Methods section to ensure reproducibility.

Response: We thank the reviewer for pointing this out. The simulations were performed using TRNSYS software. However, due to international sanctions and licensing limitations in Iran, we are not able to mention the specific name of the software in the published article. In the revised manuscript, we have tried to provide as much detail as possible regarding the modeling approach, equations, and parameters to support reproducibility. We referred to it only as “computer software” or “simulation software” for this reason.

5. The methodology relies entirely on simulation. Adding a small-scale experimental validation or comparing the simulation results with existing experimental data from the authors' previous work (Ref [14]) would increase the study's reliability.

Response: We thank the reviewer for the comment. The main objective of this study was the simulation and performance evaluation of the solar energy system. However, the Venturi-plasma pasteurization unit itself has been experimentally tested and validated in our previous studies [3,43]. We have now added a subsection at the end of the Results and Discussion section (3.11.) summarizing these experimental results.

6. The equations governing the thermal and electrical subsystems are referenced as being in Figure 4, but the text lacks a clear list of assumptions made during the simulation, such as heat loss coefficients or milk flow rate variability.

Response: Thank you for this comment. A new subsection has been added in Section 2.7 of the Materials and Methods to clearly summarize the main modeling assumptions and key simulation parameters used in the study.

7. The results highlight that February had the lowest useful heat production and energy delivery across all cities. There is a lack of deep analysis as to why February performed worse than January, which typically has lower solar altitude in the northern hemisphere.

Response: We thank the reviewer for this valuable observation. Although January typically has a lower solar altitude, the simulation results indicated that February had the lowest solar radiation receipt, useful heat gain (Qu), and energy delivered to the load (Ql) in all three cities. This appears to be primarily caused by higher cloud cover and atmospheric conditions in February, which reduced both direct and diffuse radiation more than the slight increase in solar altitude could compensate. Lower ambient temperatures in February also contributed to higher thermal losses. We have expanded the discussion in section 3.5.1 to elaborate on these climatic factors.

8. The solar fraction is noted to be "moderate" (0.036–0.32). The results would benefit from a sensitivity analysis showing how increasing the collector area or storage tank size might improve this fraction, as current values suggest a heavy (up to 96%) reliance on auxiliary heating in some scenarios.

Response: We thank the reviewer for this valuable and constructive comment. We agree that the solar fraction (0.036–0.32) is moderate and indicates a relatively high reliance on auxiliary heating (up to 96% in some months). This range is reasonable for the current fixed system size designed for a small 30-cow farm (600 l/day), but can certainly be improved. In the revised manuscript, we have added a sentence in the Conclusion section acknowledging this point and recommending that future studies increase the collector area or storage tank volume to achieve a higher solar fraction.

9. Although the abstract claims the system is "cost-effective," the results section lacks a formal economic analysis (e.g., Levelized Cost of Energy or Payback Period) to support this statement.

Response: Thanks for your benefit **comment. We acknowledge that the term “cost-effective” in the Abstract was used qualitatively, referring to the potential of the system to reduce dependence on fossil fuels and grid electricity in remote areas, rather than based on a formal economic analysis (LCOE or payback period). A detailed economic evaluation was beyond the scope of the current study, which primarily focused on technical feasibility and energetic performance simulation across different climates. In the revised manuscript, we have moderated this claim in the Abstract and Conclusion and added a clarifying sentence regarding the need for future techno-economic studies.**

10. The discussion concludes that the shift to solar energy "does not alter the microbial inactivation mechanism". However, it does not discuss how fluctuations in solar-driven power or preheating temperatures might affect the consistency of this inactivation in a real-world setting.

Response: Thanks, the reviewer for this important observation. The primary goal of the dynamic simulation was precisely to identify the optimal tilt angles, evaluate long-term performance, and minimize operational fluctuations across different climates by determining the best system configuration for each location and season. The simulation was performed with hourly time steps over a full year to ensure that the system maintains the target temperature range (40 ± 5°C) as consistently as possible. While short-term solar intermittency can occur in real conditions, the combination of thermal storage tank, stratified design, and optimized collector angles (obtained through RSM) significantly dampens these fluctuations. As shown in the results, the tank temperature remained within the acceptable deadband in the majority of operating hours. Nevertheless, we acknowledge that real-world performance may still experience some variability; therefore, in the revised manuscript we have expanded the discussion in section 3.10 to address this issue and recommend appropriate control and storage strategies.

11. The comparison of solar fractions with other studies is noted, but the discussion should further address why this system’s SF is significantly lower than the 0.57 reported in other Iranian studies (Ref [36]).

Response: Thanks for your attention. We acknowledge that the solar fraction in the present study (0.036–0.32) is lower than the value of 0.57 reported in some previous Iranian studies [36]. This difference can be mainly attributed to: (1) the smaller system scale designed for a 30-cow farm (600 L/day), (2) the relatively low operating temperature (40 ± 5°C) required for the non-thermal Venturi-plasma pasteurization process, which results in a lower thermal load compared to conventional high-temperature pasteurization, and (3) the additional electrical demand of the plasma system supplied by PV panels. In the revised manuscript, we have expanded the discussion in section 3.9 to clearly explain these reasons and compare the system characteristics with previous studies.

12. The article mentions reducing greenhouse gas emissions but does not quantify the potential CO2 savings based on the simulated auxiliary energy reductions.

Response: **We thank the reviewer for this thoughtful comment. We fully agree that quantifying CO₂ emission savings would strengthen the environmental discussion. However, we respectfully note that the primary objective of this study was to simulate the technical and energy performance of the solar-assisted pasteurization system specifically, to evaluate its feasibility across different Iranian climates and to optimize parameters such as solar fraction, collector efficiency, PV performance, and auxiliary energy demand (Qaux). A precise, quantitative estimation of CO₂ reduction would require a dedicated life-cycle assessment (LCA) with access to hourly, site-specific grid emission data (fuel mix, power plant efficiency, etc.), which was beyond the scope and resources of the present study. The statement in the manuscript regarding greenhouse gas emission reduction was intended as a qualitative claim (any renewable system displacing fossil fuel-based energy inherently reduces emissions), not as a quantified savings value. We have not claimed any specific numerical value for emission savings. To avoid speculation, we prefer not to include unsubstantiated numbers in the manuscript. Nevertheless, we acknowledge the importance of this issue and have added a sentence in the Conclusion section recommending a detailed LCA for future studies. We hope the reviewer accepts this balanced approach.**

13. While the manuscript provides a comprehensive simulation of a solar-assisted Venturi-plasma system, the theoretical framework regarding the optimization and economic viability of hybrid energy systems could be further strengthened. To improve the literature review and provide a broader context for your energy performance analysis, I recommend incorporating the following studies into your revised version: For instance, 1) the application of predictive control models in thermal management systems, such as those explored in "Controlling a House’s Air-Conditioning Using Nonlinear Model Predictive Control", offers relevant insights into achieving energy efficiency in temperature-dependent processes. 2) When discussing the performance of photovoltaic panels and the potential for the system to be "cost-effective", it would be beneficial to reference similar techno-economic assessments. Specifically, the findings in "Techno-economic viability of a hybrid power generation system that optimally integrates solar photovoltaic (PV) panels..." regarding the optimal sizing of PV arrays and the resulting reduction in levelized cost of energy (LCOE) would provide a strong comparative baseline for your claims about the economic feasibility of solar-powered units in remote areas.

Response: We sincerely thank the reviewer for this valuable and constructive suggestion. In response, the manuscript has been revised to strengthen the theoretical framework related to energy management and techno-economic considerations of hybrid solar systems. Specifically, additional discussion has been incorporated in Section 2.7 regarding the potential application of advanced predictive control strategies, including nonlinear model predictive control (NMPC), for improving thermal management and auxiliary energy optimization in solar-assisted systems. Furthermore, Section 3.9 and the Conclusion section have been expanded to include discussion of techno-economic studies on hybrid photovoltaic systems, particularly regarding optimal PV sizing and reduction of levelized cost of energy (LCOE) in remote areas. These additions provide a broader comparative framework for evaluating the economic feasibility and long-term sustainability of the proposed solar-assisted Venturi-plasma pasteurization system. The recommended references suggested by the reviewer have also been incorporated into the revised manuscript.

Reviewer #2

1. The paper is well written but the novelty needs to be highlighted against similar papers.

**Response: We appreciate the reviewer's suggestion. The novelty of the study has been further emphasized and contrasted with similar papers in the Introduction section.**

2. The abstract needs to give a summary of important results and conclusion should be shorter.

**Response:** Thank you for this helpful suggestion. We have revised the Abstract to include a more concise summary of the key findings, such as the comparative performance of the three cities and the system's impact on energy sustainability. Additionally, the Conclusion has been significantly shortened to focus on the study's primary contributions and final implications, removing redundant technical details as requested.

3. In introduction give some more references especially related to solar radiation effect.

**Response:** We sincerely thank the reviewer for this valuable suggestion. In response, additional references and discussion related to the effects of solar radiation and climatic conditions on photovoltaic and solar thermal system performance have been incorporated into the Introduction section. The revised manuscript now provides broader background regarding the influence of solar irradiance, temperature, humidity, and environmental conditions on the efficiency and stability of hybrid solar-assisted energy systems.
