# Supplementary material for: Simulation and performance analysis of a solar-assisted venturi-plasma pasteurization system for milk
Source: PLoS One. 2026 Jul 2;21(7):e0352384. doi: 10.1371/journal.pone.0352384 (PMC13327238; doi:10.1371/journal.pone.0352384)
Supplement: Peer review history

# Peer review: Decision Letter 0

**DOI:** 10.1371/journal.pone.0352384.r001

**Authors:** Soheil Mohtaram

16 Apr 2026

PONE-D-26-00284

Simulation and Performance Analysis of a Solar-Assisted Venturi-Plasma Pasteurization System for Milk

PLOS One

Dear Dr. Hosseinzadeh Samani,

Thank you for submitting your manuscript to PLOS ONE. After careful consideration, we feel that it has merit but does not fully meet PLOS ONE’s publication criteria as it currently stands. Therefore, we invite you to submit a revised version of the manuscript that addresses the points raised during the review process.

After careful evaluation of your manuscript and consideration of the reviewers’ comments, I regret to inform you that the manuscript cannot be accepted for publication in its current form. However, the reviewers and I believe that the study addresses an interesting and relevant topic, and it has the potential to make a meaningful contribution to the field. Therefore, we invite you to revise your manuscript substantially and resubmit it for further consideration.

The reviewers have raised several important concerns regarding the clarity of methodology, depth of analysis, validation of results, and overall presentation of the work. In particular, you are encouraged to:

- Provide clearer and more rigorous descriptions of the modeling and simulation approach.

- Strengthen the validation of your results, including comparison with experimental data or established benchmarks where possible.

- Improve the discussion section by offering deeper insights into the system performance and its practical implications.

- Address all reviewer comments in a detailed, point-by-point response.

- Ensure that the manuscript is revised for clarity, structure, and language quality.

Please carefully revise your manuscript in line with all comments provided and submit a detailed response-to-reviewers document explaining how each point has been addressed. Where changes have been made, please indicate them clearly in the revised manuscript.

Please submit your revised manuscript by May 30 2026 11:59PM. If you will need more time than this to complete your revisions, please reply to this message or contact the journal office at plosone@plos.org. When you're ready to submit your revision, log on to https://www.editorialmanager.com/pone/ and select the 'Submissions Needing Revision' folder to locate your manuscript file.

Please include the following items when submitting your revised manuscript:

- A letter that responds to each point raised by the academic editor and reviewer(s). You should upload this letter as a separate file labeled 'Response to Reviewers'.

- A marked-up copy of your manuscript that highlights changes made to the original version. You should upload this as a separate file labeled 'Revised Manuscript with Track Changes'.

- An unmarked version of your revised paper without tracked changes. You should upload this as a separate file labeled 'Manuscript'.

If you would like to make changes to your financial disclosure, please include your updated statement in your cover letter. Guidelines for resubmitting your figure files are available below the reviewer comments at the end of this letter.

If applicable, we recommend that you deposit your laboratory protocols in protocols.io to enhance the reproducibility of your results. Protocols.io assigns your protocol its own identifier (DOI) so that it can be cited independently in the future. For instructions see: https://journals.plos.org/plosone/s/submission-guidelines#loc-laboratory-protocols. Additionally, PLOS ONE offers an option for publishing peer-reviewed Lab Protocol articles, which describe protocols hosted on protocols.io. Read more information on sharing protocols at https://plos.org/protocols?utm_medium=editorial-email&utm_source=authorletters&utm_campaign=protocols.

As the corresponding author, your ORCID iD is verified in the submission system and will appear in the published article. PLOS supports the use of ORCID, and we encourage all coauthors to register for an ORCID iD and use it as well. Please encourage your coauthors to verify their ORCID iD within the submission system before final acceptance, as unverified ORCID iDs will not appear in the published article. *Only*  the individual author can complete the verification step; PLOS staff *cannot*  verify ORCID iDs on behalf of authors.

We look forward to receiving your revised manuscript.

Kind regards,

Dr. Soheil Mohtaram

Academic Editor

PLOS One

Journal Requirements:

When submitting your revision, we need you to address these additional requirements.

1. Please ensure that your manuscript meets PLOS ONE's style requirements, including those for file naming. The PLOS ONE style templates can be found at

https://journals.plos.org/plosone/s/file?id=wjVg/PLOSOne_formatting_sample_main_body.pdf and

https://journals.plos.org/plosone/s/file?id=ba62/PLOSOne_formatting_sample_title_authors_affiliations.pdf

2. Please note that PLOS One has specific guidelines on code sharing for submissions in which author-generated code underpins the findings in the manuscript. In these cases, we expect all author-generated code to be made available without restrictions upon publication of the work. Please review our guidelines at https://journals.plos.org/plosone/s/materials-and-software-sharing#loc-sharing-code and ensure that your code is shared in a way that follows best practice and facilitates reproducibility and reuse.

3. Thank you for stating the following financial disclosure: [The Research Council of Shahrekord University is gratefully acknowledged for the financial support to carry out this work (grant No: 01GRN1M1796), and this work is based on research funded by the Iran National Science Foundation (INSF) under project No. 4038682.].

Please state what role the funders took in the study.  If the funders had no role, please state: ""The funders had no role in study design, data collection and analysis, decision to publish, or preparation of the manuscript.""

If this statement is not correct you must amend it as needed.

Please include this amended Role of Funder statement in your cover letter; we will change the online submission form on your behalf.

4. Thank you for stating the following in the Acknowledgments Section of your manuscript:[ The Research Council of Shahrekord University is gratefully acknowledged for the financial support to carry out this work (grant No: 01GRN1M1796), and this work is based on research funded by the Iran National Science Foundation (INSF) under project No. 4038682.]

We note that you have provided funding information that is not currently declared in your Funding Statement. However, funding information should not appear in the Acknowledgments section or other areas of your manuscript. We will only publish funding information present in the Funding Statement section of the online submission form.

Please remove any funding-related text from the manuscript and let us know how you would like to update your Funding Statement. Currently, your Funding Statement reads as follows: [The Research Council of Shahrekord University is gratefully acknowledged for the financial support to carry out this work (grant No: 01GRN1M1796), and this work is based on research funded by the Iran National Science Foundation (INSF) under project No. 4038682.].

Please include your amended statements within your cover letter; we will change the online submission form on your behalf.

5. We note that your Data Availability Statement is currently as follows: [All relevant data are within the manuscript and its Supporting Information files.]

Please confirm at this time whether or not your submission contains all raw data required to replicate the results of your study. Authors must share the “minimal data set” for their submission. PLOS defines the minimal data set to consist of the data required to replicate all study findings reported in the article, as well as related metadata and methods (https://journals.plos.org/plosone/s/data-availability#loc-minimal-data-set-definition).

For example, authors should submit the following data:

- The values behind the means, standard deviations and other measures reported;

- The values used to build graphs;

- The points extracted from images for analysis.

Authors do not need to submit their entire data set if only a portion of the data was used in the reported study.

If your submission does not contain these data, please either upload them as Supporting Information files or deposit them to a stable, public repository and provide us with the relevant URLs, DOIs, or accession numbers. For a list of recommended repositories, please see https://journals.plos.org/plosone/s/recommended-repositories.

If there are ethical or legal restrictions on sharing a de-identified data set, please explain them in detail (e.g., data contain potentially sensitive information, data are owned by a third-party organization, etc.) and who has imposed them (e.g., an ethics committee). Please also provide contact information for a data access committee, ethics committee, or other institutional body to which data requests may be sent. If data are owned by a third party, please indicate how others may request data access.

6. If the reviewer comments include a recommendation to cite specific previously published works, please review and evaluate these publications to determine whether they are relevant and should be cited. There is no requirement to cite these works unless the editor has indicated otherwise.

[Note: HTML markup is below. Please do not edit.]

Reviewers' comments:

Reviewer's Responses to Questions

**Comments to the Author**

1. Is the manuscript technically sound, and do the data support the conclusions?

The manuscript must describe a technically sound piece of scientific research with data that supports the conclusions. Experiments must have been conducted rigorously, with appropriate controls, replication, and sample sizes. The conclusions must be drawn appropriately based on the data presented.

Reviewer #1: Yes

Reviewer #2: Yes

**********

2. Has the statistical analysis been performed appropriately and rigorously?

Reviewer #1: Yes

Reviewer #2: Yes

**********

3. Have the authors made all data underlying the findings in their manuscript fully available?

The PLOS Data policy requires authors to make all data underlying the findings described in their manuscript fully available without restriction, with rare exception (please refer to the Data Availability Statement in the manuscript PDF file). The data should be provided as part of the manuscript or its supporting information, or deposited to a public repository. For example, in addition to summary statistics, the data points behind means, medians and variance measures should be available. If there are restrictions on publicly sharing data—e.g. participant privacy or use of data from a third party—those must be specified.

Reviewer #1: No

Reviewer #2: Yes

**********

4. Is the manuscript presented in an intelligible fashion and written in standard English?

PLOS ONE does not copyedit accepted manuscripts, so the language in submitted articles must be clear, correct, and unambiguous. Any typographical or grammatical errors should be corrected at revision, so please note any specific errors here.

Reviewer #1: Yes

Reviewer #2: Yes

**********

5. Review Comments to the Author

Please use the space provided to explain your answers to the questions above. You may also include additional comments for the author, including concerns about dual publication, research ethics, or publication ethics. (Please upload your review as an attachment if it exceeds 20,000 characters)

Reviewer #1: 1. While the introduction identifies that previous studies focused on conventional thermal methods, it should more clearly articulate why a non-thermal "Venturi-plasma" system specifically requires solar assistance compared to other technologies.

2. The text briefly mentions "liquid-phase cold plasma" and "hydrodynamic cavitation". A more detailed explanation of why these specific non-thermal methods were chosen over other emerging technologies (e.g., Pulsed Electric Fields or UV-C) would strengthen the rationale.

3. The introduction mentions high economic costs of traditional systems but does not provide a baseline for current energy costs in Iran to better frame the "cost-effective" claim made later.

4. The paper mentions "computer software" several times for simulation (e.g., in sections 2.1 and 2.6). The specific software name (e.g., TRNSYS, MATLAB, or a custom build) should be explicitly stated in the Materials and Methods section to ensure reproducibility.

5. The methodology relies entirely on simulation. Adding a small-scale experimental validation or comparing the simulation results with existing experimental data from the authors' previous work (Ref [14]) would increase the study's reliability.

6. The equations governing the thermal and electrical subsystems are referenced as being in Figure 4, but the text lacks a clear list of assumptions made during the simulation, such as heat loss coefficients or milk flow rate variability.

7. The results highlight that February had the lowest useful heat production and energy delivery across all cities. There is a lack of deep analysis as to why February performed worse than January, which typically has lower solar altitude in the northern hemisphere.

8. The solar fraction is noted to be "moderate" (0.036–0.32). The results would benefit from a sensitivity analysis showing how increasing the collector area or storage tank size might improve this fraction, as current values suggest a heavy (up to 96%) reliance on auxiliary heating in some scenarios.

9. Although the abstract claims the system is "cost-effective," the results section lacks a formal economic analysis (e.g., Levelized Cost of Energy or Payback Period) to support this statement.

10. The discussion concludes that the shift to solar energy "does not alter the microbial inactivation mechanism". However, it does not discuss how fluctuations in solar-driven power or preheating temperatures might affect the consistency of this inactivation in a real-world setting.

11. The comparison of solar fractions with other studies is noted, but the discussion should further address why this system’s SF is significantly lower than the 0.57 reported in other Iranian studies (Ref [36]).

12. The article mentions reducing greenhouse gas emissions but does not quantify the potential CO2 savings based on the simulated auxiliary energy reductions.

13. While the manuscript provides a comprehensive simulation of a solar-assisted Venturi-plasma system, the theoretical framework regarding the optimization and economic viability of hybrid energy systems could be further strengthened. To improve the literature review and provide a broader context for your energy performance analysis, I recommend incorporating the following studies into your revised version: For instance, 1) the application of predictive control models in thermal management systems, such as those explored in "Controlling a House’s Air-Conditioning Using Nonlinear Model Predictive Control", offers relevant insights into achieving energy efficiency in temperature-dependent processes. 2) When discussing the performance of photovoltaic panels and the potential for the system to be "cost-effective", it would be beneficial to reference similar techno-economic assessments. Specifically, the findings in "Techno-economic viability of a hybrid power generation system that optimally integrates solar photovoltaic (PV) panels..." regarding the optimal sizing of PV arrays and the resulting reduction in levelized cost of energy (LCOE) would provide a strong comparative baseline for your claims about the economic feasibility of solar-powered units in remote areas.

Reviewer #2: The paper is well written but the novelty needs to be highlighted against similar papers. The abstract needs to give a summary of important results and conclusion should be shorter. In introduction give some more references especially related to solar radiation effect

**********

6. PLOS authors have the option to publish the peer review history of their article (what does this mean?). If published, this will include your full peer review and any attached files.

If you choose “no”, your identity will remain anonymous but your review may still be made public.

**Do you want your identity to be public for this peer review?**  For information about this choice, including consent withdrawal, please see our Privacy Policy.

Reviewer #1: **Yes:** AHMAD H. SABRY

Reviewer #2: No

**********

[NOTE: If reviewer comments were submitted as an attachment file, they will be attached to this email and accessible via the submission site. Please log into your account, locate the manuscript record, and check for the action link "View Attachments". If this link does not appear, there are no attachment files.]

To ensure your figures meet our technical requirements, please review our figure guidelines: https://journals.plos.org/plosone/s/figures

You may also use PLOS’s free figure tool, NAAS, to help you prepare publication quality figures: https://journals.plos.org/plosone/s/figures#loc-tools-for-figure-preparation.

NAAS will assess whether your figures meet our technical requirements by comparing each figure against our figure specifications.

# Author comment: Author response to Decision Letter 1

**DOI:** 10.1371/journal.pone.0352384.r002

12 May 2026

Response to the honorable referee of the paper entitled:

Simulation and Performance Analysis of a Solar-Assisted Venturi-Plasma Pasteurization System for Milk

Thanking the comments and proposed amendments of the honorable referees, the answer to the referred points is mentioned in the separation of different parts of the paper as follows:

Reviewer #1:

1. While the introduction identifies that previous studies focused on conventional thermal methods, it should more clearly articulate why a non-thermal "Venturi-plasma" system specifically requires solar assistance compared to other technologies.

Response: Thanks for this constructive comment. In response to the reviewer’s comment, in introduction section we emphasize that although the Venturi–plasma pasteurization system is principally non thermal, a mild solar-assisted preheating (up to 35–40 °C) significantly enhances hydrodynamic performance and microbial reduction without compromising the non thermal nature. Moreover, photovoltaic panels supply the required electrical energy for plasma generation and control units through renewable sources, enabling fully off grid operation. This integration allows feasible deployment in remote dairy farms where conventional energy supply is limited, thus underscoring the necessity and novelty of solar assistance in this system.

2. The text briefly mentions "liquid-phase cold plasma" and "hydrodynamic cavitation". A more detailed explanation of why these specific non-thermal methods were chosen over other emerging technologies (e.g., Pulsed Electric Fields or UV-C) would strengthen the rationale.

Response: thanks for your benefit comment. We chose the Venturi-plasma system because it is a non-thermal method with a strong synergy between its components. In the Venturi tube, pressure drops and velocity changes create a high turbulence flow. This turbulent environment helps to inactivate microorganisms and creates cavitation, which weakens the microbial cell walls. By combining this with liquid-phase plasma which is an innovative approach the plasma is generated directly inside the milk. While the plasma provides disinfection, the turbulence from the Venturi helps to distribute the generated radicals throughout the liquid. This makes the treatment more uniform and effective than UV-C (which cannot penetrate milk) or PEF (which faces fouling issues).

3. The introduction mentions high economic costs of traditional systems but does not provide a baseline for current energy costs in Iran to better frame the "cost-effective" claim made later.

Response: Thank you for this valuable comment. In this study, our primary focus was on the technical feasibility and energy performance of the proposed solar-assisted system. The term “cost-effective” was mainly used in the context of remote and off-grid dairy farms (equivalent to a 30-cow farm), where access to reliable grid electricity is limited or nonexistent. In such areas, any reduction in dependence on fossil fuels or diesel generators can significantly lower operational costs. However, we acknowledge that a detailed economic analysis (payback period or life-cycle cost) was not performed in the current work. We have revised the manuscript by moderating the economic claims and adding a clarifying sentence in the Introduction and Conclusion to better reflect the scope of the study.

4. The paper mentions "computer software" several times for simulation (e.g., in sections 2.1 and 2.6). The specific software name (e.g., TRNSYS, MATLAB, or a custom build) should be explicitly stated in the Materials and Methods section to ensure reproducibility.

Response: We thank the reviewer for pointing this out. The simulations were performed using TRNSYS software. However, due to international sanctions and licensing limitations in Iran, we are not able to mention the specific name of the software in the published article. In the revised manuscript, we have tried to provide as much detail as possible regarding the modeling approach, equations, and parameters to support reproducibility. We referred to it only as “computer software” or “simulation software” for this reason.

5. The methodology relies entirely on simulation. Adding a small-scale experimental validation or comparing the simulation results with existing experimental data from the authors' previous work (Ref [14]) would increase the study's reliability.

Response: We thank the reviewer for the comment. The main objective of this study was the simulation and performance evaluation of the solar energy system. However, the Venturi-plasma pasteurization unit itself has been experimentally tested and validated in our previous studies [3,43]. We have now added a subsection at the end of the Results and Discussion section (3.11.) summarizing these experimental results.

6. The equations governing the thermal and electrical subsystems are referenced as being in Figure 4, but the text lacks a clear list of assumptions made during the simulation, such as heat loss coefficients or milk flow rate variability.

Response: Thank you for this comment. A new subsection has been added in Section 2.7 of the Materials and Methods to clearly summarize the main modeling assumptions and key simulation parameters used in the study.

7. The results highlight that February had the lowest useful heat production and energy delivery across all cities. There is a lack of deep analysis as to why February performed worse than January, which typically has lower solar altitude in the northern hemisphere.

Response: We thank the reviewer for this valuable observation. Although January typically has a lower solar altitude, the simulation results indicated that February had the lowest solar radiation receipt, useful heat gain (Qu), and energy delivered to the load (Ql) in all three cities. This appears to be primarily caused by higher cloud cover and atmospheric conditions in February, which reduced both direct and diffuse radiation more than the slight increase in solar altitude could compensate. Lower ambient temperatures in February also contributed to higher thermal losses. We have expanded the discussion in section 3.5.1 to elaborate on these climatic factors.

8. The solar fraction is noted to be "moderate" (0.036–0.32). The results would benefit from a sensitivity analysis showing how increasing the collector area or storage tank size might improve this fraction, as current values suggest a heavy (up to 96%) reliance on auxiliary heating in some scenarios.

Response: We thank the reviewer for this valuable and constructive comment. We agree that the solar fraction (0.036–0.32) is moderate and indicates a relatively high reliance on auxiliary heating (up to 96% in some months). This range is reasonable for the current fixed system size designed for a small 30-cow farm (600 l/day), but can certainly be improved. In the revised manuscript, we have added a sentence in the Conclusion section acknowledging this point and recommending that future studies increase the collector area or storage tank volume to achieve a higher solar fraction.

9. Although the abstract claims the system is "cost-effective," the results section lacks a formal economic analysis (e.g., Levelized Cost of Energy or Payback Period) to support this statement.

Response: Thanks for your benefit comment. We acknowledge that the term “cost-effective” in the Abstract was used qualitatively, referring to the potential of the system to reduce dependence on fossil fuels and grid electricity in remote areas, rather than based on a formal economic analysis (LCOE or payback period). A detailed economic evaluation was beyond the scope of the current study, which primarily focused on technical feasibility and energetic performance simulation across different climates. In the revised manuscript, we have moderated this claim in the Abstract and Conclusion and added a clarifying sentence regarding the need for future techno-economic studies.

10. The discussion concludes that the shift to solar energy "does not alter the microbial inactivation mechanism". However, it does not discuss how fluctuations in solar-driven power or preheating temperatures might affect the consistency of this inactivation in a real-world setting.

Response: Thanks, the reviewer for this important observation. The primary goal of the dynamic simulation was precisely to identify the optimal tilt angles, evaluate long-term performance, and minimize operational fluctuations across different climates by determining the best system configuration for each location and season. The simulation was performed with hourly time steps over a full year to ensure that the system maintains the target temperature range (40 ± 5°C) as consistently as possible. While short-term solar intermittency can occur in real conditions, the combination of thermal storage tank, stratified design, and optimized collector angles (obtained through RSM) significantly dampens these fluctuations. As shown in the results, the tank temperature remained within the acceptable deadband in the majority of operating hours. Nevertheless, we acknowledge that real-world performance may still experience some variability; therefore, in the revised manuscript we have expanded the discussion in section 3.10 to address this issue and recommend appropriate control and storage strategies.

11. The comparison of solar fractions with other studies is noted, but the discussion should further address why this system’s SF is significantly lower than the 0.57 reported in other Iranian studies (Ref [36]).

Response: Thanks for your attention. We acknowledge that the solar fraction in the present study (0.036–0.32) is lower than the value of 0.57 reported in some previous Iranian studies [36]. This difference can be mainly attributed to: (1) the smaller system scale designed for a 30-cow farm (600 L/day), (2) the relatively low operating temperature (40 ± 5°C) required for the non-thermal Venturi-plasma pasteurization process, which results in a lower thermal load compared to conventional high-temperature pasteurization, and (3) the additional electrical demand of the plasma system supplied by PV panels. In the revised manuscript, we have expanded the discussion in section 3.9 to clearly explain these reasons and compare the system characteristics with previous studies.

12. The article mentions reducing greenhouse gas emissions but does not quantify the potential CO2 savings based on the simulated auxiliary energy reductions.

Response: We thank the reviewer for this thoughtful comment. We fully agree that quantifying CO₂ emission savings would strengthen the environmental discussion. However, we respectfully note that the primary objective of this study was to simulate the technical and energy performance of the solar-assisted pasteurization system specifically, to evaluate its feasibility across different Iranian climates and to optimize parameters such as solar fraction, collector efficiency, PV performance, and auxiliary energy demand (Qaux). A precise, quantitative estimation of CO₂ reduction would require a dedicated life-cycle assessment (LCA) with access to hourly, site-specific grid emission data (fuel mix, power plant efficiency, etc.), which was beyond the scope and resources of the present study. The statement in the manuscript regarding greenhouse gas emission reduction was intended as a qualitative claim (any renewable system displacing fossil fuel-based energy inherently reduces emissions), not as a quantified savings value. We have not claimed any specific numerical value for emission savings. To avoid speculation, we prefer not to include unsubstantiated numbers in the manuscript. Nevertheless, we acknowledge the importance of this issue and have added a sentence in the Conclusion section recommending a detailed LCA for future studies. We hope the reviewer accepts this balanced approach.

13. While the manuscript provides a comprehensive simulation of a solar-assisted Venturi-plasma system, the theoretical framework regarding the optimization and economic viability of hybrid energy systems could be further strengthened. To improve the literature review and provide a broader context for your energy performance analysis, I recommend incorporating the following studies into your revised version: For instance, 1) the application of predictive control models in thermal management systems, such as those explored in "Controlling a House’s Air-Conditioning Using Nonlinear Model Predictive Control", offers relevant insights into achieving energy efficiency in temperature-dependent processes. 2) When discussing the performance of photovoltaic panels and the potential for the system to be "cost-effective", it would be beneficial to reference similar techno-economic assessments. Specifically, the findings in "Techno-economic viability of a hybrid power generation system that optimally integrates solar photovoltaic (PV) panels..." regarding the optimal sizing of PV arrays and the resulting reduction in levelized cost of energy (LCOE) would provide a strong comparative baseline for your claims about the economic feasibility of solar-powered units in remote areas.

Response: We sincerely thank the reviewer for this valuable and constructive suggestion. In response, the manuscript has been revised to strengthen the theoretical framework related to energy management and techno-economic considerations of hybrid solar systems. Specifically, additional discussion has been incorporated in Section 2.7 regarding the potential application of advanced predictive control strategies, including nonlinear model predictive control (NMPC), for improving thermal management and auxiliary energy optimization in solar-assisted systems. Furthermore, Section 3.9 and the Conclusion section have been expanded to include discussion of techno-economic studies on hybrid photovoltaic systems, particularly regarding optimal PV sizing and reduction of levelized cost of energy (LCOE) in remote areas. These additions provide a broader comparative framework for evaluating the economic feasibility and long-term sustainability of the proposed solar-assisted Venturi-plasma pasteurization system. The recommended references suggested by the reviewer have also been incorporated into the revised manuscript.

Reviewer #2

1. The paper is well written but the novelty needs to be highlighted against similar papers.

Response: We appreciate the reviewer's suggestion. The novelty of the study has been further emphasized and contrasted with similar papers in the Introduction section.

2. The abstract needs to give a summary of important results and conclusion should be shorter.

Response: Thank you for this helpful suggestion. We have revised the Abstract to include a more concise summary of the key findings, such as the comparative performance of the three cities and the system's impact on energy sustainability. Additionally, the Conclusion has been significantly shortened to focus on the study's primary contributions and final implications, removing redundant technical details as requested.

3. In introduction give some more references especially related to solar radiation effect.

Response: We sincerely thank the reviewer for this valuable suggestion. In response, additional references and discussion related to the effects of solar radiation and climatic conditions on photovoltaic and solar thermal system performance have been incorporated into the Introduction section. The revised manuscript now provides broader background regarding the influence of solar irradiance, temperature, humidity, and environmental conditions on the efficiency and stability of hybrid solar-assisted energy systems.

Attachment Submitted filename: answer to reviewer.docx

# Peer review: Decision Letter 1

**DOI:** 10.1371/journal.pone.0352384.r003

**Authors:** Soheil Mohtaram

9 Jun 2026

Simulation and Performance Analysis of a Solar-Assisted Venturi-Plasma Pasteurization System for Milk

PONE-D-26-00284R1

Dear Dr. Bahram Hosseinzadeh Samani,

We’re pleased to inform you that your manuscript has been judged scientifically suitable for publication and will be formally accepted for publication once it meets all outstanding technical requirements.

Within one week, you’ll receive an e-mail detailing the required amendments. When these have been addressed, you’ll receive a formal acceptance letter and your manuscript will be scheduled for publication.

An invoice will be generated when your article is formally accepted. Please note, if your institution has a publishing partnership with PLOS and your article meets the relevant criteria, all or part of your publication costs will be covered. Please make sure your user information is up-to-date by logging into Editorial Manager at Editorial Manager® and clicking the ‘Update My Information' link at the top of the page. For questions related to billing, please contact billing support.

If your institution or institutions have a press office, please notify them about your upcoming paper to help maximize its impact. If they’ll be preparing press materials, please inform our press team as soon as possible -- no later than 48 hours after receiving the formal acceptance. Your manuscript will remain under strict press embargo until 2 pm Eastern Time on the date of publication. For more information, please contact onepress@plos.org.

Kind regards,

Soheil Mohtaram

Academic Editor

PLOS One

-->Review Comments to the Author

Please use the space provided to explain your answers to the questions above. You may also include additional comments for the author, including concerns about dual publication, research ethics, or publication ethics. (Please upload your review as an attachment if it exceeds 20,000 characters)

Reviewer #1: Thank you, no more comments

Reviewer #2: The revision of paper can be accepted now . The authors answered well The concerns so I am happy to accept it

# Editor report: Acceptance letter

**DOI:** 10.1371/journal.pone.0352384.r004

**Authors:** Soheil Mohtaram

PONE-D-26-00284R1

PLOS One

Dear Dr. Hosseinzadeh Samani,

I'm pleased to inform you that your manuscript has been deemed suitable for publication in PLOS One. Congratulations! Your manuscript is now being handed over to our production team.

At this stage, our production department will prepare your paper for publication. This includes ensuring the following:

* All references, tables, and figures are properly cited

* All relevant supporting information is included in the manuscript submission,

* There are no issues that prevent the paper from being properly typeset

You will receive further instructions from the production team, including instructions on how to review your proof when it is ready. Please keep in mind that we are working through a large volume of accepted articles, so please give us a few days to review your paper and let you know the next and final steps.

Lastly, if your institution or institutions have a press office, please let them know about your upcoming paper now to help maximize its impact. If they'll be preparing press materials, please inform our press team within the next 48 hours. Your manuscript will remain under strict press embargo until 2 pm Eastern Time on the date of publication. For more information, please contact onepress@plos.org.

You will receive an invoice from PLOS for your publication fee after your manuscript has reached the completed accept phase. If you receive an email requesting payment before acceptance or for any other service, this may be a phishing scheme. Learn how to identify phishing emails and protect your accounts at https://explore.plos.org/phishing.

If we can help with anything else, please email us at customercare@plos.org.

Thank you for submitting your work to PLOS ONE and supporting open access.

Kind regards,

PLOS ONE Editorial Office Staff

on behalf of

Dr. Soheil Mohtaram

Academic Editor

PLOS One
